# A new herrerasaurian dinosaur from the Upper Triassic Upper Maleri Formation of south-central India

**DOI:** 10.1098/rsos.250081

**Published:** 2025-05-07

**Authors:** Martín D. Ezcurra, Maurício Silva Garcia, Fernando E. Novas, Rodrigo Temp Müller, Federico L. Agnolín, Sankar Chatterjee

**Affiliations:** ^1^ Sección Paleontología de Vertebrados, Museo Argentino de Ciencias Naturales 'Bernardino Rivadavia', Ciudad Autónoma de Buenos Aires, Argentina; ^2^ School of Geography, Earth and Environmental Sciences, University of Birmingham, Birmingham, UK; ^3^ Consejo Nacional de Investigaciones Científicas y Técnicas, Ciudad Autónoma de Buenos Aires, Argentina; ^4^ Programa de Pós-Graduação em Biodiversidade Animal, Universidade Federal de Santa Maria, Santa Maria, Rio Grande do Sul, Brazil; ^5^ Centro de Apoio à Pesquisa Paleontológica da Quarta Colônia, Universidade Federal de Santa Maria, São João do Polêsine, Rio Grande do Sul, Brazil; ^6^ Departamento de Ciencias Naturales y Antropología, Fundación de Historia Natural ‘Félix de Azara’, Universidad Maimónides, Ciudad Autónoma de Buenos Aires, Argentina; ^7^ Laboratorio de Anatomia Comparada y Evolución de los Vertebrados, Museo Argentino de Ciencias Naturales ‘Bernardino Rivadavia’, Ciudad Autónoma de Buenos Aires, Argentina; ^8^ Department of Museum, Texas Tech University, Lubbock, TX, USA

**Keywords:** Dinosauria, Saurischia, Herrerasauria, Norian, Late Triassic, Pranhita-Godavari

## Abstract

Some of the oldest known dinosaurs and the first faunas numerically dominated by them are documented in the Upper Triassic–Lower Jurassic-aged Gondwana formations exposed in the Pranhita-Godavari Valley of south-central and east-central India. The Upper Maleri Formation of the Pranhita-Godavari Basin preserves an early-middle Norian dinosaur assemblage numerically dominated by sauropodomorph dinosaurs, including at least two nominal species. However, the preliminary report of a herrerasaurian dinosaur specimen indicates that this assemblage of south-central Gondwana was more taxonomically diverse. Here, we describe and compare in detail the anatomy and assess the taxonomy and phylogenetic relationships of the Upper Maleri herrerasaurian specimen. A unique combination of character states present in this specimen allows the erection of the new genus and species *Maleriraptor kuttyi*. Updated quantitative phylogenetic analyses focused on early dinosauriforms recovered *Maleriraptor kuttyi* as a member of Herrerasauria outside of the South American clade Herrerasauridae. *Maleriraptor kuttyi* fills a temporal gap between the Carnian South American herrerasaurids and the younger middle Norian–Rhaetian herrerasaurs of North America. *Maleriraptor kuttyi* shows the first evidence that herrerasaurs survived also in Gondwana the early Norian tetrapod turnover that resulted in the global extinction of the rhynchosaurs.

## 1. Introduction

**Figure 1.**
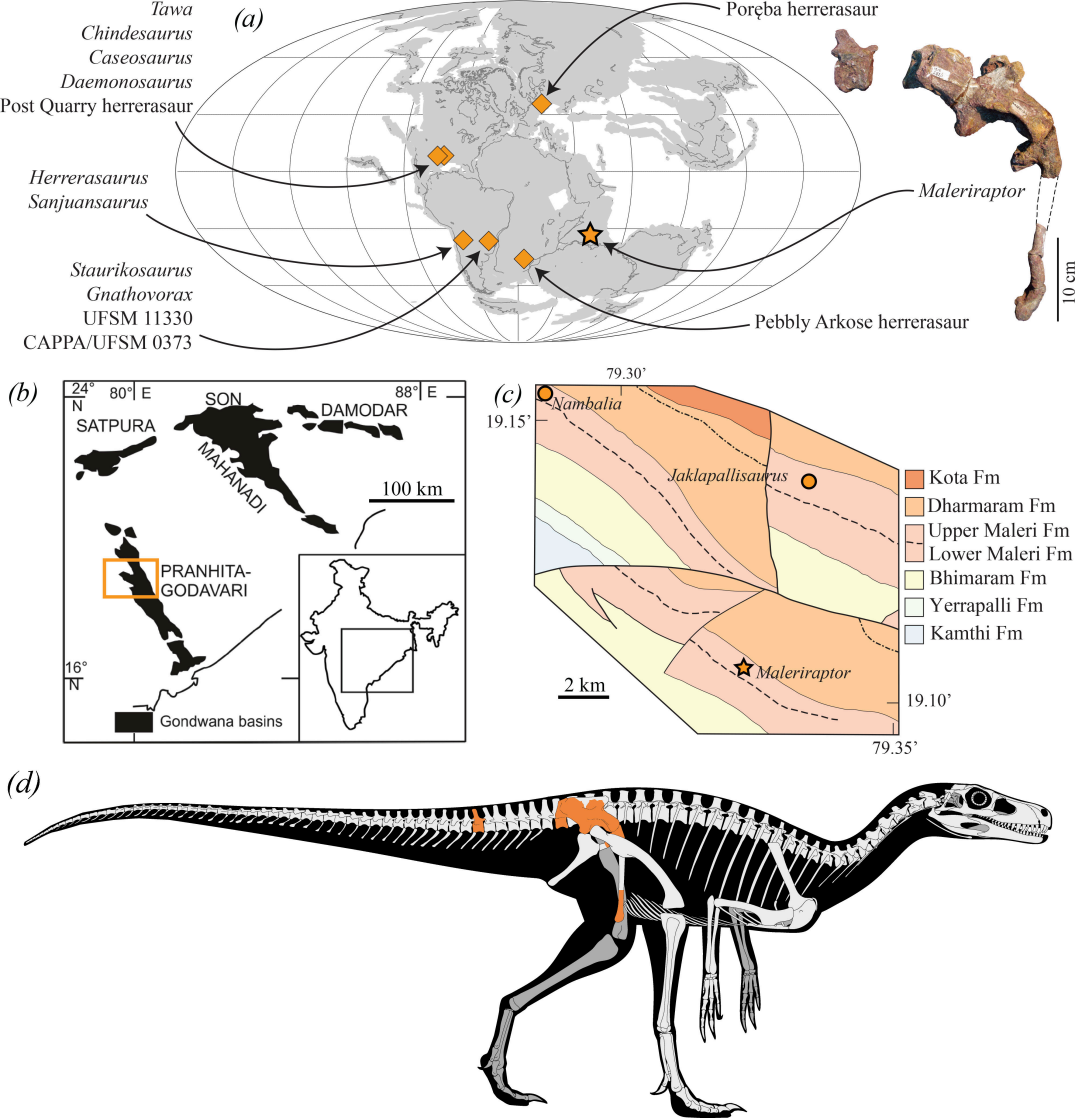
Geographic and stratigraphic occurrence and preserved bones of *Maleriraptor kuttyi*. (*a*) Palaeomap of the Late Triassic depicting the occurrences of the herrerasaurs. (*b*) Overview of the Gondwana basins in India, with the Pranhita-Godavari valley highlighted (modified from [32]). (*c*) Detailed geological map of a portion of the Pranhita-Godavari valley indicating the type localities of the nominal dinosaur species of the Upper Maleri Formation. (*d*) Reconstruction of the skeleton of *Maleriraptor kuttyi* (drawn by M.S.G.) showing the preserved bones in colour. (*b*) Modified from Sengupta *et al*. [33] and (*c*) modified from Kutty & Sengupta [34].

Herrerasaurs represent the oldest radiation of predatory dinosaurs [1–7]. Until recently, their record was unambiguously restricted to four nominal species of the middle Carnian–lowermost Norian beds of the Ischigualasto Formation of northwestern Argentina (*Herrerasaurus ischigualastensis* and *Sanjuansaurus gordilloi*) and the lower portion of the Candelária Sequence of the Santa Maria Supersequence of southern Brazil (*Staurikosaurus pricei* and *Gnathovorax cabreirai*) (*ca* 233−229 Ma [8,9]). These species are bipedal forms ranging from 1.2 to 6 m in total length [7,10]. In particular, *Herrerasaurus ischigualastensis* is the most abundant dinosaur in the lower third of the stratigraphic sequence of the Ischigualasto Formation at the Hoyada de Ischigualasto locality [11]. The possible presence of herrerasaurs outside of South America was first suggested in the mid−1990s with the description of *Chindesaurus bryansmalli* from middle-upper Norian levels of the Chinle Formation of North America [2,12]. Although some subsequent quantitative analyses recovered *Chindesaurus bryansmalli* as a herrerasaur (e.g. [13–19]), other studies do not (e.g. [20–24]). The probable presence of herrerasaurs in North America was also indicated by the description of a specimen from the middle Norian of the Dockum Group that was interpreted as an early saurischian similar to *Staurikosaurus pricei* [25] or as a herrerasaurid [26]. Recent quantitative phylogenetic analyses bolster the distribution of herrerasaurs beyond South America with the identification of other species as members of this clade, namely *Saltopus elginensis* (middle Carnian–earliest Norian of Scotland) and *Caseosaurus crosbyensis* (early Norian of the United States) in one analysis [18] and of *Tawa hallae* (middle-late Norian of the United States) and *Daemonosaurus chauliodus* (Rhaetian of the United States) in other analysis [7]. The herrerasaurian affinities of *Saltopus elginensis* have not been recovered in subsequent analyses and seem unlikely, and this species is probably a non-saurischian dinosauriform [6,19,27,28]. The taxonomically broader Herrerasauria, including *Tawa hallae*, *Chindesaurus bryansmalli* and *Daemonosaurus chauliodus*, was also recently found by another phylogenetic analysis [19]. In addition, more fragmentary, unnamed herrerasaur records have been reported from other Upper Triassic units of North America (Dockum Group, [26]), Poland [29], Zimbabwe [30] and possibly India [7,31] (figure 1*a*).

The vast majority of the Triassic dinosaur remains reported in India come from the Pranhita-Godavari Basin in south-central India [32,35]. This basin is an approximately 7 km thick succession of sedimentary rocks deposited from the late Carboniferous/early Permian to the Cretaceous [36]. The Triassic dinosaur-bearing units of the Pranhita-Godavari Basin are the Lower Maleri (middle-late Carnian), Upper Maleri (early Norian) and Lower Dharmaram (middle Norian–Rhaetian) formations [7,35]. The putative herrerasaur record of India is based on a partial postcranium (ISIR 282; figure 1*a*) collected in the Upper Maleri Formation (figure 1*b*,*c*). This specimen was first mentioned in an abstract by Novas *et al*. [31] as a specimen that resembled herrerasaurs in the presence of a vertically oriented pubis and the absence of a brevis fossa on the ilium. Subsequently, ISIR 282 was preliminarily described and figured by Novas *et al*. [35] and these authors included it in a quantitative phylogenetic analysis focused on early dinosaurs. ISIR 282 was recovered in a polytomy also composed of Ornithischia, Theropoda, Sauropodomorpha and all the herrerasaurian species-level taxa, leading Novas *et al*. [35] to refer ISIR 282 to an indeterminate genus and species of Dinosauriformes. More recently, the herrerasaur affinities of ISIR 282 have been noticed again by Novas *et al*. [7], but without further discussion.

The Upper Maleri Formation that yielded the remains of ISIR 282 is particularly relevant to shed light on the early evolution of dinosaurs because it preserves a dinosaur assemblage slightly younger than the initial Carnian radiation of the group (i.e. shortly after the end of the rhynchosaurian dominance) [37]. Earliest Norian dinosaur assemblages are very scarce worldwide, being mostly restricted to the above-mentioned Indian unit and the upper portion of the Candelária Sequence of the Santa Maria Supersequence of Brazil (slightly younger than *ca* 225 Myr; [8]). The first dinosaur discoveries in the Upper Maleri Formation were reported by Kutty & Sengupta [34] and were identified as small, early diverging sauropodomorphs (cf. *Massospondylus* sp. in Kutty *et al*. [38]; aff. *Guaibasaurus* in Kutty *et al*. [39]). However, these specimens were described several years later, including ISIR 282, an unnamed early sauropodomorph, and two nominal species that were erected at that time, the non-sauropodiform sauropodomorphs *Nambalia roychowdhurii* and *Jaklapallisaurus asymmetricus* [35]. All these dinosaurs were described briefly and with a limited number of illustrations as part of a broader paper documenting the dinosaur-bearing assemblages of the Upper Maleri, and its overlying unit, the Lower Dharmaram formations. Among the dinosaurs of the Upper Maleri Formation, only *Jaklapallisaurus asymmetricus* has been described in detail [37]. Thus, the aim of this paper is to describe in detail and reassess the taxonomy and phylogenetic relationships of ISIR 282.

## 2. Material and methods

### 2.1. Phylogenetic analyses

The phylogenetic relationships of ISIR 282 were tested using two independent data matrices. The first matrix is that used by Ezcurra *et al*. [40], which is the latest modification of the matrix originally published by Nesbitt *et al*. [14] and that was iteratively modified by subsequent authors (see Ezcurra [41] for a short discussion of the genealogy of this matrix: ‘*Tawa* matrix’). This matrix is focused on early saurischian relationships, and here it was modified with the scoring of ISIR 282 and TTU-P10082, a few scoring changes, and the addition of two characters (see electronic supplementary material). The modified version of the data matrix is composed of 389 characters scored across 61 active terminals (electronic supplementary material: ‘Ezcurra_et_al_data_matrix_MaleriraptorB.tnt’). The following 32 characters were considered as ordered following Ezcurra *et al*. [40]: 9, 18, 30, 67, 128, 129, 174, 184, 197, 207, 213, 219, 231, 236, 248, 253, 254, 273, 329, 343, 345, 347, 349, 354, 366, 371, 374, 377−379, 383 and 384. The second matrix is that used by Garcia *et al*. [19] and it is a modification of the data matrix published by Norman *et al*. [24]. This dataset has been used recently to explore herrerasaurian interrelationships [19]. Here, we scored ISIR 282 and TTU-P10082, modified a character and changed a few scorings. The modified version of the data matrix is composed of 292 characters scored across 77 active terminals (electronic supplementary material: ‘Ezcurra_et_al_data_matrix_Maleriraptor.tnt’). The following 30 characters were considered as ordered following Garcia et al. [19]: 4, 13, 18, 25, 63, 82, 83, 84, 87, 89, 109, 142, 166, 174, 175, 184, 186, 190, 201, 203, 205, 209, 212, 225, 235, 236, 239, 250, 256 and 291.

Both datasets were analysed under implied weighting maximum parsimony in the program TNT version 1.6 [42]. This decision of weighting against homoplasy follows the results of the analyses of Goloboff *et al*. [43] (based on simulations) and Ezcurra [41] (based on empirical data), in which implied weighting outperformed equal weighting in topological accuracy and stability, respectively. Each dataset was analysed using ranges of concavity constant values (*k*) [41]. The Ezcurra *et al*. [40] data matrix was analysed with *k*-values between 5 and 8 following the suggestion of Ezcurra [41] for a matrix with the number of terminals used here (but excluding *k*-values of 3 and 4 because Ezcurra [41] found that these analyses underperformed other *k*-values in the genealogy of the ‘*Tawa* matrix’). The Garcia *et al*. [19] data matrix was analysed with *k*-values between 3 and 10 following the suggestion of Ezcurra [41] for a matrix between 70 and 80 terminals.

The tree searches involved 1000 replications of Wagner trees (with random addition sequence) followed by tree bisection and reconnection (TBR) branch swapping (holding 10 trees per replicate). The shortest trees obtained were then subjected to a final round of TBR branch swapping. Zero-length branches among any of the recovered most parsimonious trees (MPTs) were collapsed (rule 3 of Swofford & Begle [44] and Coddington & Scharff [45]). All the trees were rooted with *Erythrosuchus africanus* in the case of the Ezcurra *et al*. [40] data matrix and *Euparkeria capensis* in the case of the Garcia *et al*. [19] data matrix. Homoplasy indices for each analysis under the different *k*-values were calculated with the ‘STATSb.run’ script [46]. Group supports were quantified using no-zero weight symmetric resampling analyses, using 1000 pseudo-replications (each with 10 replications of Wagner trees + TBR) and reporting both absolute and group present/contradicted (GC) frequencies. Finally, a global strict consensus tree (GSCT) was generated from all the MPTs found in all the analyses using the different *k*-values. Similarly, absolute and GC resampling frequencies were calculated from all the resampling trees recovered using the different *k*-values and plotted on the branches of the GSCT. These analyses were implemented in one custom script written for TNT and named ‘treeSearches_protocol.run’ (see [47,48]; electronic supplementary material). This script, ‘STATSb.run’, the data matrix files and a subfolder called ‘output’ (it has to be created manually in Windows) should all be in the same working directory. The ‘treeSearches_protocol.run’ script needs the following four arguments that allow the user to customize the analysis: (i) the name of the matrix file without the ‘.tnt’ extension, (ii) the lower limit of the *k*-values range, (iii) the upper limit of the *k*-values range, and (iv) the number of pseudo-replications of the resampling analyses. Hence, to reproduce the analyses conducted here, the script should be run as follows in TNT (GUI users should deactivate the ‘Preview trees’ option before running the script): ‘run treeSearches_protocol.run Ezcurra_et_al_data_matrix_Maleriraptor 3 10 1000;’ (for the modified version of the Garcia *et al*. [19] data matrix) and ‘run treeSearches_protocol.run Ezcurra_et_al_data_matrix_Maleriraptor_B 5 8 1000;’ (for the modified version of the Ezcurra *et al*. [40] data matrix).

### 2.2. Institutional abbreviations

**CAPPA/UFSM**, Centro de Apoio à Pesquisa Paleontológica da Quarta Colônia, Universidade Federal de Santa Maria, São João do Polêsine, Brazil; **ISI**, Indian Statistical Institute, Kolkata, India; **MB**, Museum für Naturkunde and Leibniz-Institut für Evolutions- und Biodiversitätsforschung, Berlin, Germany; **MCP**, Museu de Ciências e Tecnología, Pontificia Universidade Catolica, Porto Alegre, Brazil; **MCZ**, Museum of Comparative Zoology, Harvard University, Cambridge, USA; **PEFO**, Petrified Forest National Park, Arizona, USA; **PVL**, Paleontología de Vertebrados, Instituto ‘Miguel Lillo’, San Miguel de Tucumán, Argentina; **PVSJ**, División de Paleontología de Vertebrados, Instituto y Museo de Ciencias Naturales y Universidad Nacional de San Juan, San Juan, Argentina; **TTU**, Museum of Texas Tech University, Lubbock, Texas, USA; **UMMP**, University of Michigan Museum of Paleontology, Ann Arbor, Michigan, USA.

### 2.3. Nomenclatural acts

This published work and the nomenclatural acts it contains have been registered in ZooBank, the online registration system for the International Code of Zoological Nomenclature. The ZooBank Life Science Identifiers (LSIDs) and the associated information can be viewed through any standard web browser by appending the LSID to the prefix ‘http://zoobank.org/’. The LSID for this publication is: urn:lsid:zoobank.org:pub:B633935E-A5FC−4E53−9D24−760836356BC9.

## 3. Systematic palaeontology

Dinosauria Owen, 1842 [49] [Langer *et al*., 2020] [50]

Saurischia Seeley, 1887 [51] [Gauthier *et al*., 2020] [52]

Herrerasauria Galton, 1985 [53] *sensu* Langer, 2004 [54]

*Maleriraptor* gen. nov.

**LSID**: urn:lsid:zoobank.org:act:F6599525-01E5-489B-B3D1-45CE16A6F03A

**Type and only species**: *Maleriraptor kuttyi* sp. nov.

**Etymology**: The genus name is derived from the Upper Maleri Formation, in which the holotype and only known specimen was collected, and the Greek word *raptor*, thief, which is an ending usually used for predatory dinosaur genera.

**Diagnosis**: As for the type and only known species.

*Maleriraptor kuttyi* sp. nov.

**Figure 2.**
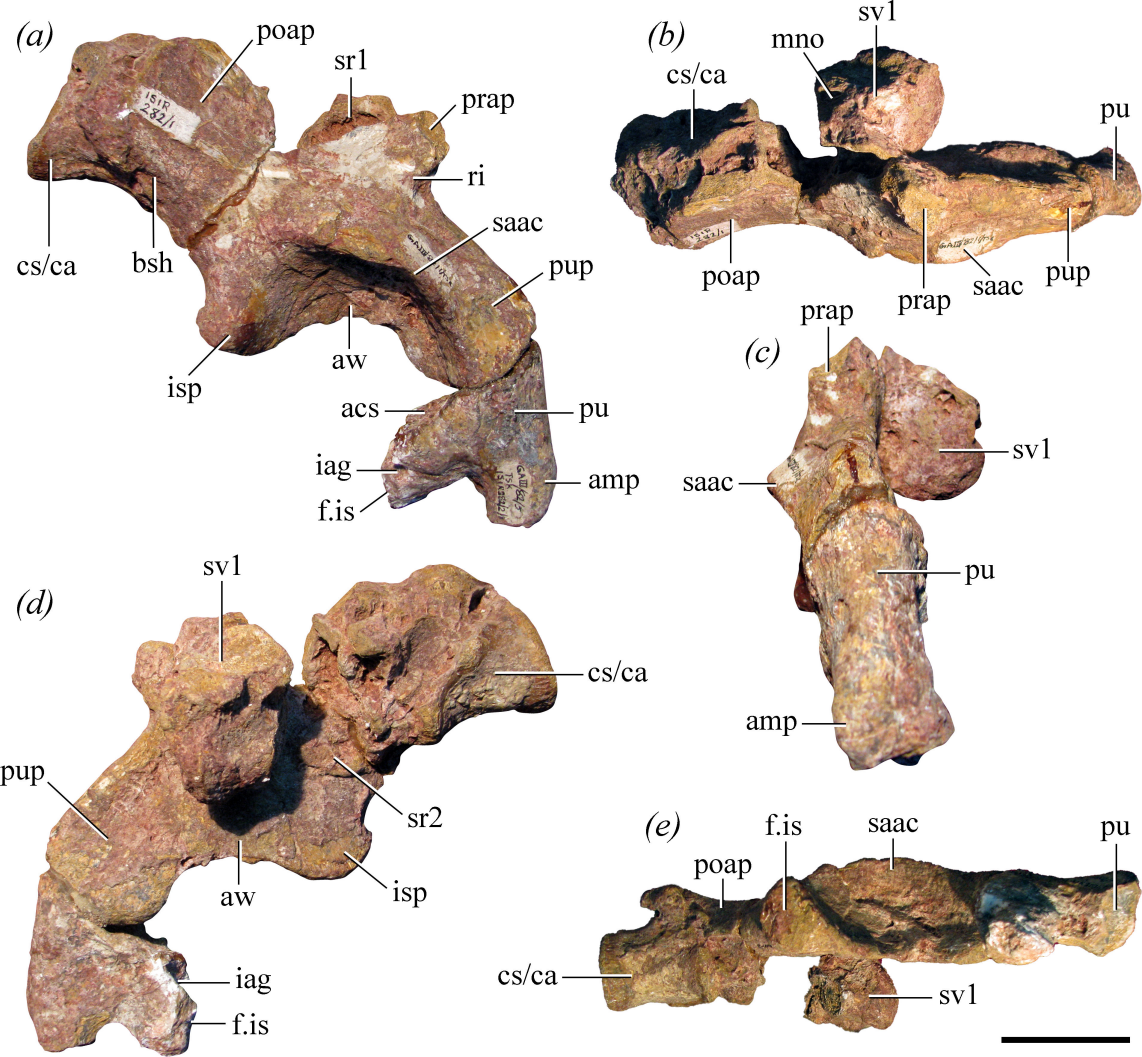
Articulated sacral and probable first caudal vertebrae, right ilium and proximal end of right pubis of the holotype of *Maleriraptor kuttyi* (ISIR 282) in (*a*) lateral, (*b*) dorsal, (*c*) anterior, (*d*) medial and (*e*) ventral views. Abbreviations: acs, acetabular surface; amp, ambiens process; aw, acetabular wall; bsh, brevis shelf; cs/ca, caudosacral or first caudal vertebra; f.is, facet for ischium; iag, ischio-acetabular groove; isp, ischial peduncle; mno, median notch; poap, postacetabular process; prap, preacetabular process; pu, pubis; pup, pubic peduncle; ri, ridge; saac, supraacetabular crest; sr1, sacral rib 1; sr2, sacral rib 2; sv1, sacral vertebra 1. Scale bar equals 5 cm.

**Figure 3.**
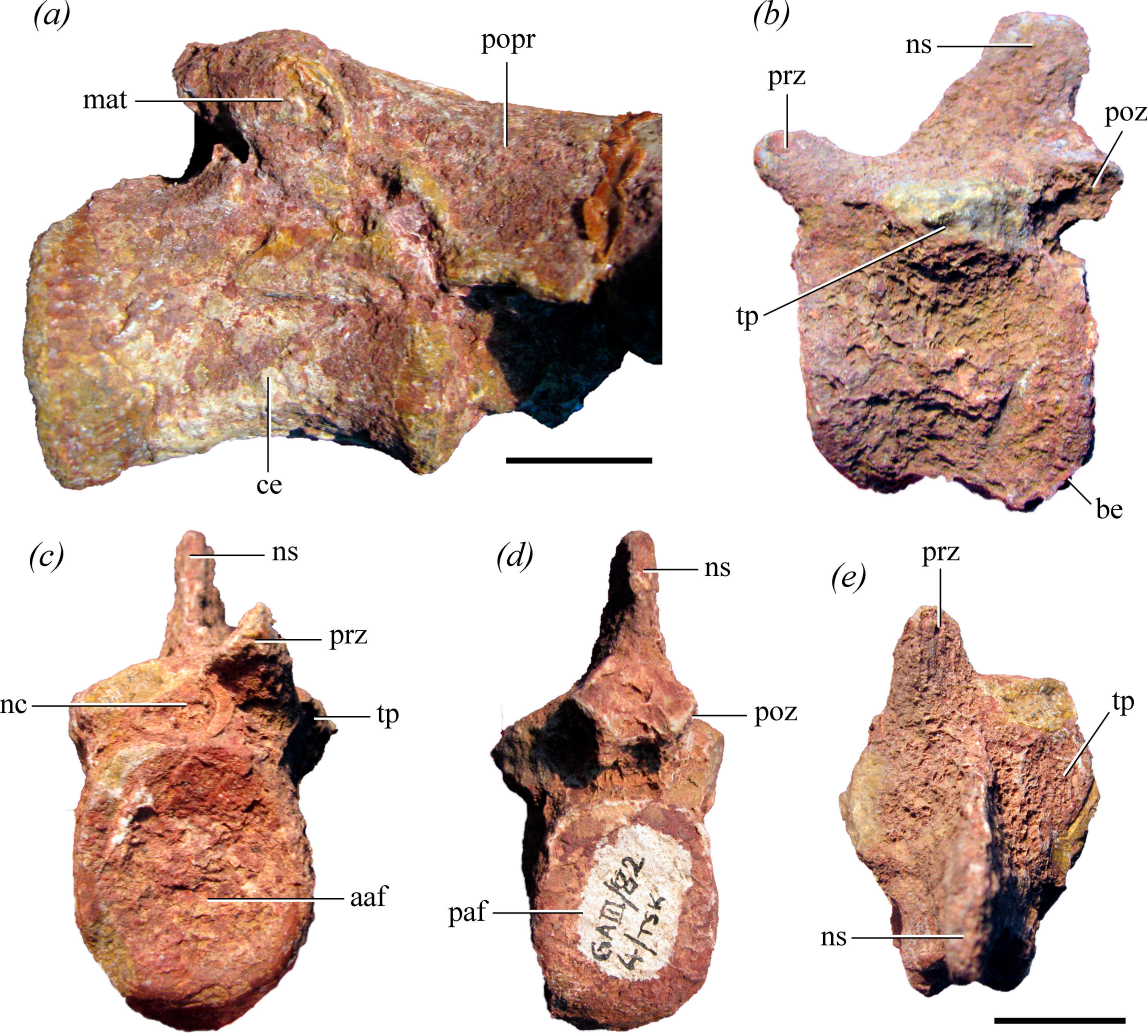
Caudal vertebrae of the holotype of *Maleriraptor kuttyi* (ISIR 282). (*a*) Probable first caudal vertebra and (b–e) another anterior caudal vertebra in (*a*) right ventrolateral, (*b*) left lateral, (*c*) anterior, (*d*) posterior and (*e*) dorsal views. Abbreviations: aaf, anterior articular surface; be, bevelled surface; ce, centrum; mat, matrix; nc, neural canal; ns, neural spine; paf, posterior articular surface; popr, postacetabular process; poz, postzygapophysis; prz, prezygapophysis; tp, transverse process. Scale bar equals 2 cm.

**Figure 4.**
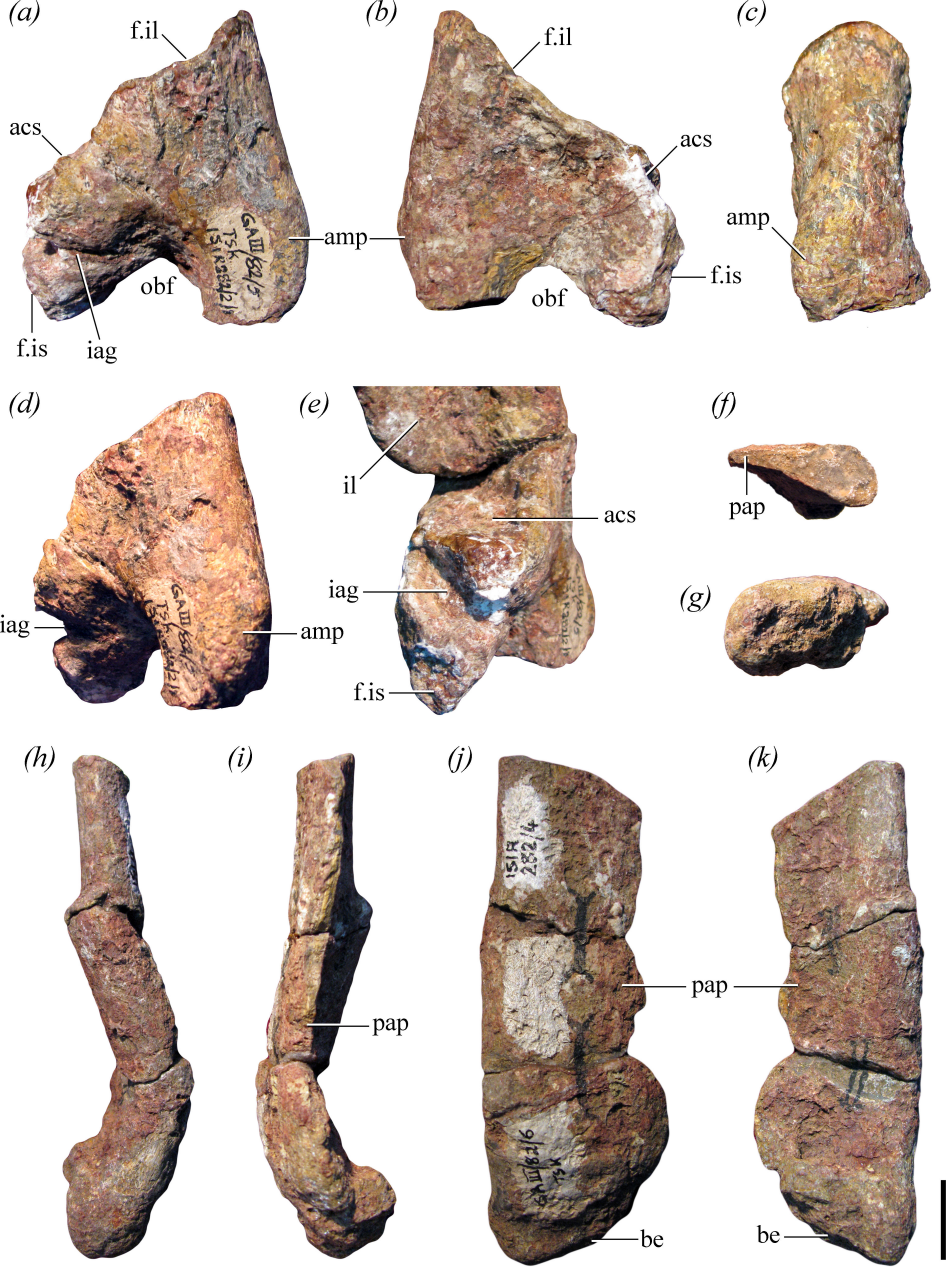
Right pubis of the holotype of *Maleriraptor kuttyi* (ISIR 282). (a–e) Proximal end and (f–k) distal portion in (*a,h*) lateral, (*b,i*) medial, (*c,j*) anterior, (*d*) anterolateral, (*e*) posterolateral, (*f*) proximal, (*g*) distal and (*k*) posterior views. Abbreviations: acs, acetabular surface; amp, ambiens process; be, bevelling; f.il, facet for ilium; f.is, facet for ischium; iag, ischio-acetabular groove; il, ilium; obf, obturator foramen, pap, pubic apron. Scale bar equals 2 cm.

**Figure 5.**
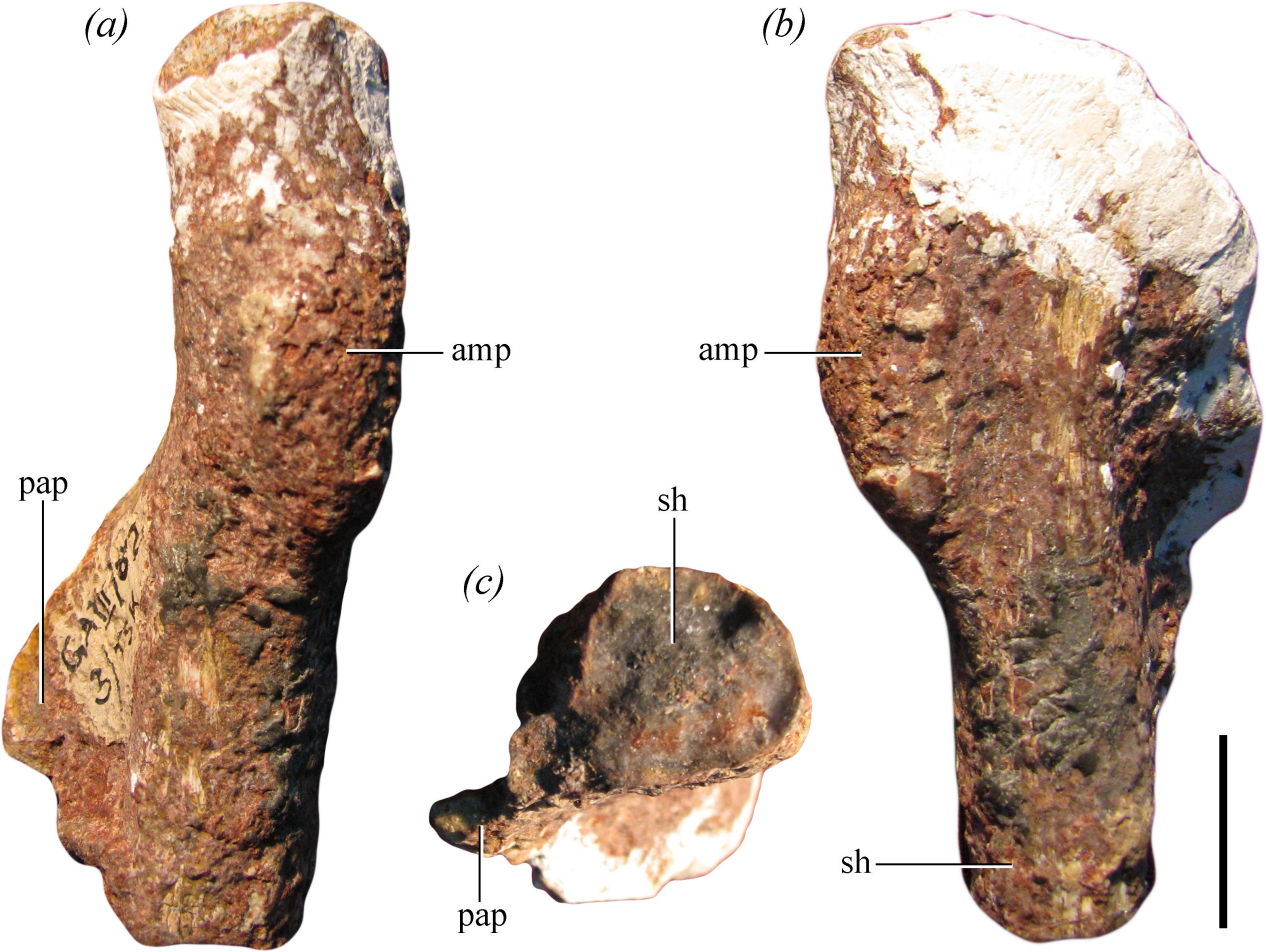
Proximal end of left pubis of the holotype of *Maleriraptor kuttyi* (ISIR 282) in (*a*) anterior and slightly medial, (*b*) lateral and slightly posterior and (*c*) proximal views. Abbreviations: amp, ambiens process; sh, shaft; pap, pubic apron. Scale bar equals 2 cm.

**Figure 6.**
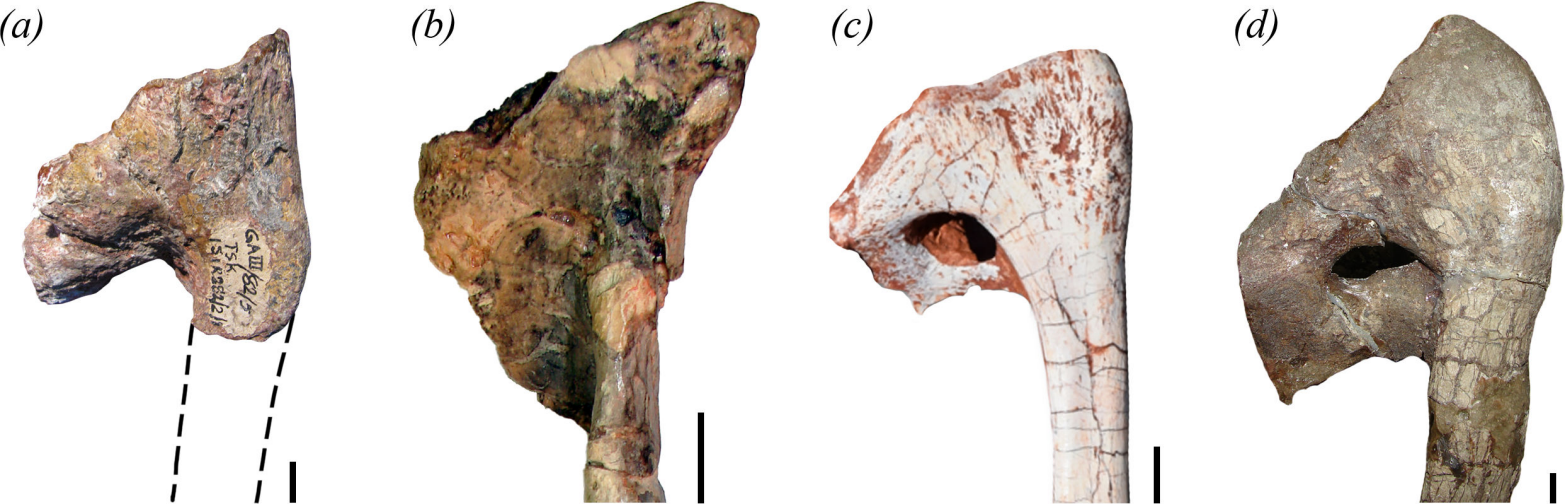
Proximal end of right pubes in lateral view of the holotype of (*a*) *Maleriraptor kuttyi* (ISIR 282), (*b*) *Staurikosaurus pricei* (MCZ 1669), (*c*) *Gnathovorax cabreirai* (CAPPA/UFSM 0009), and (*d*) *Herrerasaurus ischigualastensis* (PVL 2566). Scale bars equal 1 cm.

Figure 1*a*, figures 2–5, figure 6*a*

**LSID**: urn:lsid:zoobank.org:act:1DDBD2D9-AC18−4695-A0D5−7091248A39EA

**Holotype**: ISIR 282, a first primordial sacral vertebra with its right rib and the base of the left rib, the distalmost portion of a right second sacral rib, a caudosacral or first caudal vertebra, an anterior caudal vertebra, right ilium, proximal and distal ends of right pubis and proximal end of left pubis (figure 1*a*, figures 2−5).

**Stratigraphic and geographic occurrence**: Upper Maleri Formation (early Norian, Late Triassic), Pranhita-Godavari Valley, around 1 km south of the Annaram village, south-central India (figure 1*b*,*c*). ISIR 282 was collected more than 40 years ago and we lack georeferenced data.

**Etymology**: The species name commemorates the late T. S. Kutty, who discovered the holotype and co-authored its preliminary description with some of the authors of this study (M.D.E., F.E.N., S.C.).

**Diagnosis**: *Maleriraptor kuttyi* is an early diverging dinosauriform that differs from all other Triassic archosaurs in the presence of the following unique combination of character states (autapomorphy indicated with an asterisk): centrum posterior to the second primordial sacral vertebra longer than each of the primordial sacral centra; ilium with a postacetabular process shorter than 0.6 times the length between the pubic and ischial peduncles, absence of brevis fossa, lateral rugosity of the iliac postacetabular process restricted to its posterior-most tip*; pubis with ventrally directed shaft; and pubis with a very poorly developed distal anteroposterior expansion.

## 4. Results

### 4.1. Description

#### 4.1.1. Sacral vertebrae and ribs

The sacrum of *Maleriraptor kuttyi* is represented by, at least, a partial sacral vertebra with ribs and the distal end of another right sacral rib (figure 2). The right sacral ribs are preserved in articulation with the ilium, and it can be determined based on their position with respect to this bone that they belong to the two primordial sacral elements (see e.g. [55]). The vertebra immediately posterior to the second sacral rib could represent a caudosacral element [35]. However, this vertebra is displaced from its natural position and the centrum is preserved attached to the medial surface of the postacetabular process of the ilium, without room for a sacral rib. Indeed, the transverse processes of this vertebra are missing. Thus, it cannot be determined unambiguously whether this vertebra was integrated into the sacrum, with a rib contacting the ilium, or if it was not. The sacrum of *Gnathovorax cabreirai* (CAPPA/UFSM 0009), *Herrerasaurus ischigualastensis* [56], *Sanjuansaurus gordilloi* [3] and probably *Tawa hallae* [14] lacks a caudosacral vertebra.

The first primordial sacral element lacks the posterior portion of its centrum, the neural spine and most of the left rib (figure 2: sr1, sv1). The centrum is anteroposteriorly longer than dorsoventrally tall. The postzygapophyses are partially preserved, and thus, its centrum should not have been much longer than preserved. Although its anterior articular surface is covered with matrix, it is clear that it is wider than tall, contrasting with the taller-than-wide anterior surface of the first sacral vertebra of *Herrerasaurus ischigualastensis* [56]. The first sacral centrum of *Maleriraptor kuttyi* is well transversely compressed around mid-length, resembling *Herrerasaurus ischigualastensis* [56] and *Sanjuansaurus gordilloi* (PVSJ 605). The ventral surface of the centrum is continuously convex, without a keel or groove, as in *Sanjuansaurus gordilloi* (PVSJ 605) and *Herrerasaurus ischigualastensis* (PVL 2566). The bases of the postzygapophyses are separated by a deep median notch. The right sacral rib is robust and contacts the base of the preacetabular process, as in the first primordial sacral rib of other archosauriforms [57]. It possesses a well-developed posterodorsal component that extends posteriorly close to the dorsal margin of the iliac blade and results in a C-shaped iliac articular surface, as occurs in several other early saurischians [55]. The ventral region of the first sacral rib, if preserved, is covered with matrix and the vertebra, and it cannot be determined if it was as dorsoventrally tall as in the herrerasaurids *Gnathovorax cabreirai* (CAPPA/UFSM 0009), *Herrerasaurus ischigualastensis* and *Staurikosaurus pricei* [1,55].

Only the distalmost region of the right second primordial sacral rib is preserved (figure 2*d*: sr2). It is firmly attached to the medial surface of the ilium dorsal to the ischiadic penduncle. It cannot be determined its morphology more posteriorly because it is covered by the vertebra posterior to the second sacral rib.

#### 4.1.2. Caudosacral or first caudal vertebra

The centrum of this vertebra is longer than what is estimated for the length of the first primordial sacral centrum (figures 2 and 3*a*). This condition contrasts with that of *Gnathovorax cabreirai* (CAPPA/UFSM 0009), *Herrerasaurus ischigualastensis* (PVL 2566) and *Sanjuansaurus gordilloi* [3], in which the first caudal centrum is shorter than the two primordial sacral centra. The posterior articular surface of the centrum is flat and oval, dorsoventrally taller than broad. The centrum is slightly transversely compressed around mid-length but at a lower degree than in the first primordial sacral centrum. The ventral surface of the centrum is continuously convex, without a keel or groove, as in *Gnathovorax cabreirai* (CAPPA/UFSM 0009), *Herrerasaurus ischigualastensis* (PVL 2566), *Sanjuansaurus gordilloi* [3] and *Staurikosaurus pricei* [58]. The base of the transverse process is anteroposteriorly long, extending along slightly more than half the length of the centrum.

#### 4.1.3. Anterior caudal vertebra

This vertebra is fairly complete, lacking most of the transverse processes and the right prezygapophysis (figure 3*b*–*e*). The height of the neural spine along its main axis is 0.66 times the height of the posterior articular surface of the centrum. This proportionally low neural spine suggests that it probably does not belong to the first five caudal vertebrae because the anteriormost caudal elements of *Gnathovorax cabreirai* [6], *Herrerasaurus ischigualastensis* [56] and *Sanjuansaurus gordilloi* [3] have considerably taller neural spines. The centrum is slightly longer than tall, as in *Gnathovorax cabreirai* (CAPPA/UFSM 0009) and the fourth caudal centrum of *Staurikosaurus pricei* [58], but contrasting with the strongly anteroposteriorly compressed anterior caudal centra of *Herrerasaurus ischigualastensis* [56]. The posterior margin of the centrum is slightly more ventrally extended than the anterior one, which is a common feature among the anterior caudal vertebrae of archosaurs. The posteroventral surface of the centrum is bevelled for articulation with a haemal arch. The anterior and posterior articular surfaces of the centrum are oval, being taller than wide. The anterior articular surface is slightly concave and the posterior surface is mostly flat. There is no trace of the neurocentral suture. Only the bases of the transverse processes are preserved and they are anteroposteriorly positioned at the level of mid-length of the centrum. The base of the transverse process is sub-oval in cross-section, as in *Herrerasaurus ischigualastensis* (PVL 2566) and *Staurikosaurus pricei* [58], but contrasting with the dorsoventrally compressed transverse processes of *Gnathovorax cabreirai* (CAPPA/UFSM 0009) and *Sanjuansaurus gordilloi* [3]. The orientation of the transverse processes cannot be determined. The prezygapophysis is short and slightly dorsally oriented. The postzygapophyses are also very short, extending slightly beyond the level of the centrum. The zygapophyses are anteroposteriorly oriented and their articular surfaces slant at an angle close to 45°. There is no hyposphene-hypantrum articulation. The base of the neural spine extends along most of the length of the neural arch, being developed from the base of the prezygapophyses up to the posterior end of the neural arch, between both postzygapophyses. The neural spine is relatively low and posterodorsally oriented. This contrasts with the vertical neural spines of the first three caudal vertebrae of *Gnathovorax cabreirai* [6], *Herrerasaurus ischigualastensis* and *Staurikosaurus pricei* [1], but the preserved anterior caudal vertebra of *Maleriraptor kuttyi* is probably posterior to this region of the tail. The distal end of the neural spine lacks an expansion.

#### 4.1.4. Ilium

Most of the right ilium is preserved, only lacking the anterior end of the preacetabular process and most of the dorsal margin of the iliac blade (figure 2). A well-developed and thick ridge extends along the anterior margin of the base of the preacetabular process, resembling the condition in silesaurids and other early saurischians [57]. The postacetabular process is anteroposteriorly very short, representing 0.57 times the length of the acetabulum and resembling the condition in *Gnathovorax cabreirai* [6], *Herrerasaurus ischigualastensis* and *Staurikosaurus pricei* [1]. By contrast, the length of the postacetabular process is 0.90 times that of the acetabulum in *Caseosaurus crosbyensis* (UMMP 8870) and higher than 1.00 times in *Tawa hallae* [14]. The posterior end of the postacetabular process of *Maleriraptor kuttyi* is rounded. The lateral surface of the posterior end of the postacetabular process lacks the strongly laterally raised rugosity present in *Herrerasaurus ischigualastensis* (PVL 2566), *Caseosaurus crosbyensis* (UMMP 8870), *Chindesaurus bryansmalli* [23], *Gnathovorax cabreirai* [6], the Post Quarry herrerasaur [25,26], the Pivetta herrerasaur [19] and several other early dinosauriforms (e.g. *Silesaurus opolensis*: [59]; *Saturnalia tupiniquim*: [60]; *Chromogisaurus novasi*: [61]). The brevis fossa is absent in *Maleriraptor kuttyi*, as in South American herrerasaurids [1,6], *Caseosaurus crosbyensis* (UMMP 8870), *Chindesaurus bryansmalli* ([12]; PEFO 10395), *Tawa hallae* [14], the Post Quarry herrerasaur [25], the Pivetta herrerasaur [19] and some non-dinosaurian avemetatarsalians, such as *Lagerpeton chanarensis* [62] and *Lagosuchus talampayensis* [63]. A faint and very short brevis shelf is present, not connected to the supraacetabular crest, resembling the herrerasaurid condition [1].

The supraacetabular crest is conspicuous, but transversely narrow, contrasting with the better laterally projected crest of herrerasaurids [6,56,64] and several eusaurischians (e.g. *Guaibasaurus candelariensis*: [65]; *Saturnalia tupiniquim*: MCP 3845-PV; *Eoraptor lunensis*: PVSJ 512; *Liliensternus liliensterni*: MB R. 2175). On the other hand, the lateral development of the supraacetabular crest of *Maleriraptor kuttyi* closely resembles that of *Caseosaurus crosbyensis* (UMMP 8870). The supraacetabular crest extends over the pubic peduncle, but it does not reach the distal end of the peduncle, as in *Caseosaurus crosbyensis* (UMMP 8870) and *Chindesaurus bryansmalli* [23]. The pubic peduncle is very long and also anteroposteriorly wide, closely resembling the overall morphology of *Herrerasaurus ischigualastensis* [56], *Staurikosaurus pricei* [64] and *Caseosaurus crosbyensis* (UMMP 8870). There is no sign of fusion between the pubic peduncle and the pubis. On the other hand, both structures are strongly fused in the holotype of *Gnathovorax cabreirai* (CAPPA/UFSM 0009). The medial surface of the base of the pubic peduncle is flat, contrasting with the presence of a shelf-like medial prominence that houses the facet for the anteroventral corner of the first primordial sacral rib in *Caseosaurus crosbyensis* (UMMP 8870). The ischiadic peduncle is very short, being considerably less ventrally extended than the pubic peduncle, closely resembling the condition in *Staurikosaurus pricei* [64], *Tawa hallae* [14] and *Caseosaurus crosbyensis* (UMMP 8870). The ischiadic peduncle also has a considerably more restricted articular surface than the pubic peduncle. The ischial articular facet is triangular in ventral view, with a slightly rounded posterior border and an anteromedial apex. There is an incipient posterior projection at the posteroventral corner of the ischiadic peduncle. Although the acetabular wall is not fully open, it is more perforated than in *Saturnalia tupiniquim* [60], *Guaibasaurus candelariensis* [65,66] and *Chromogisaurus novasi* [61], resembling the degree of perforation of *Herrerasaurus ischigualastensis* ([56]; PVL 2566) and *Gnathovorax cabreirai* (CAPPA/UFSM 0009).

#### 4.1.5. Pubis

The proximal and distal ends of the right pubis (figure 4) and the proximal end of the left element, without the iliac articulation (figure 5), are preserved. The base of the shaft of the pubis of *Maleriraptor kuttyi* is strongly posteriorly inflected and indicates that the shaft was approximately ventrally directed, resembling the condition in South American herrerasaurids (figure 6). The orientation of the pubic shaft of *Maleriraptor kuttyi* contrasts with the slightly anteroventrally oriented pubic shaft of *Tawa hallae* [14]. The proximal end of the pubis of *Staurikosaurus pricei* (figure 6*b*, MCZ 1669) has a concave anterior margin in lateral view, whereas the posterior inflection of the pubis of *Maleriraptor kuttyi* (figure 6*a*) and *Herrerasaurus ischigualastensis* (figure 6*d*) results in a convex anterior margin and a straight margin in *Gnathovorax cabreirai* (figure 6*c*).

The pubic facet for articulation with the ilium is anteroposteriorly short, and posterior to it, there is a non-articular surface that forms part of the acetabulum. Between this surface and the ischiadic peduncle, there is a broad and deep ischio-acetabular groove (figure 4: iag), as in the silesaurid *Eucoelophysis baldwini* [67] and the sauropodomorph *Saturnalia tupiniquim* [60]. This groove is ventrally bowed along its posteromedial to anterolateral extension and opens anteriorly into the obturator foramen. The tubercle for the insertion of the *M. ambiens* is mound-like and moderately developed, as in *Gnathovorax cabreirai* (CAPPA/UFSM 0009), *Herrerasaurus ischigualastensis* (PVL 2566), and *Sanjuansaurus gordilloi* [3]. The ambiens process of *Maleriraptor kuttyi* is positioned level with the obturator foramen, as in *Herrerasaurus ischigualastensis* (PVL 2566), but differing from the more proximally positioned process of *Sanjuansaurus gordilloi* [3] and *Gnathovorax cabreirai* [6]. In cross-section, the proximal end of the shaft is comma-shaped, being convex anteriorly and concave posteriorly, and anteroposteriorly thicker laterally than medially.

A plate-like, broad bone is identified here as the distal end of the right pubis (figure 4*f*–*k*) because of its rounded and thick putative lateral margin, which thins towards a lamina interpreted as the pubic apron. The pubic apron is narrow, with a width of *ca* 4 cm close to the distal end of the bone. The pubis is only very poorly posteriorly expanded distally, contrasting with the strongly developed pubic boot present in *Herrerasaurus ischigualastensis* [68], *Sanjuansaurus gordilloi* [3], *Staurikosaurus pricei* [64], *Gnathovorax cabreirai* [6], *Tawa hallae* [14] and the Post Quarry herrerasaur [25,26]. The distal end of the bone is oval, transversely broader than anteroposteriorly deep, in distal view. The pubic apron does not reach the distal end of the bone, resulting in a median subtriangular gap between both pubes in anterior and posterior views.

### 4.2. Phylogenetic results

**Figure 7.**
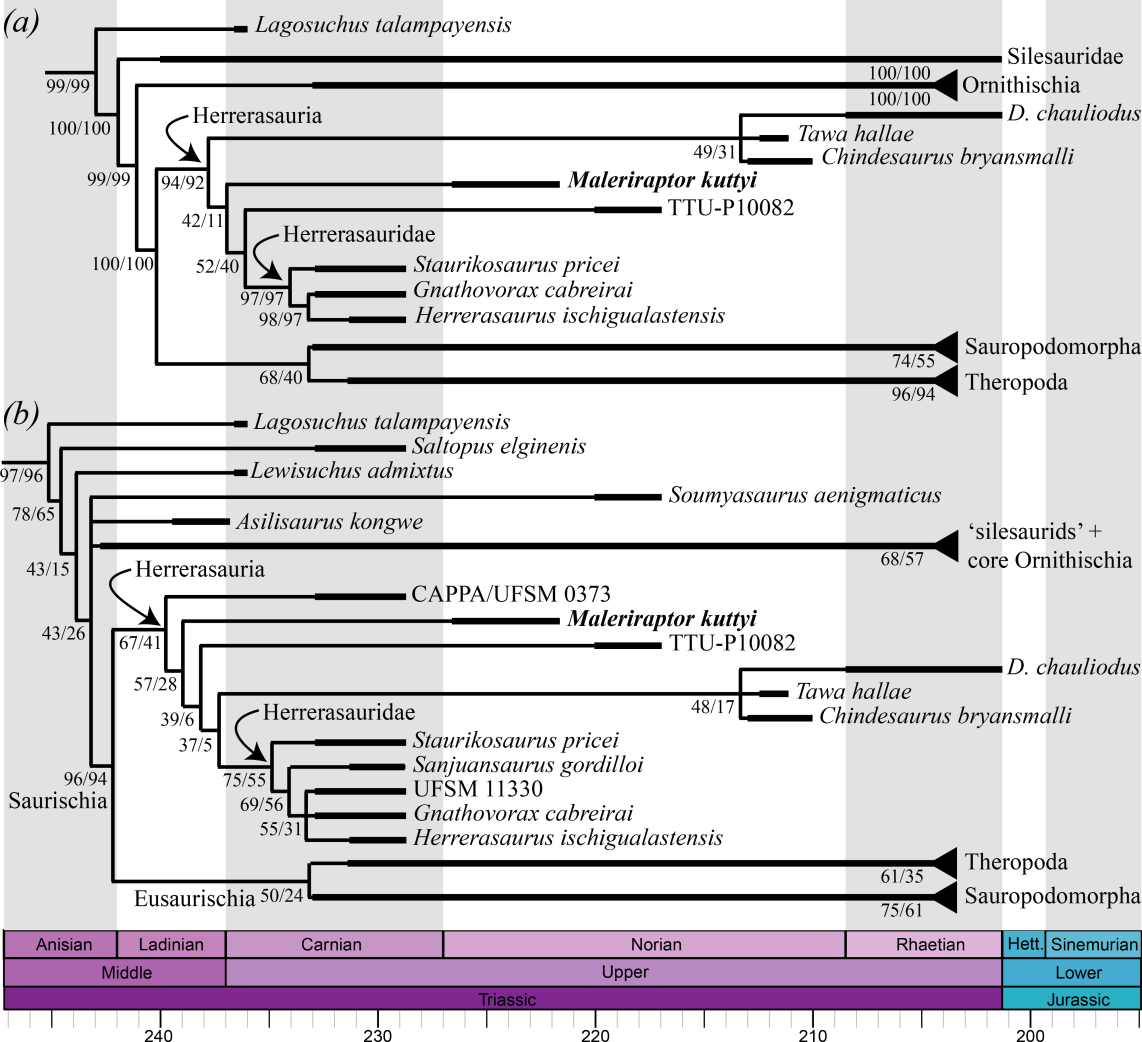
Phylogenetic relationships of *Maleriraptor kuttyi*. (*a*) Time-calibrated strict consensus subtree of the modified Garcia *et al*. [19] matrix analysed under implied weighting (concavity constant value = 10) and (*b*) time-calibrated global strict consensus subtree of the modified Ezcurra *et al*. [40] matrix analysed under implied weighting (concavity constant values = 5−8). Values below each branch represent absolute (left) and GC (right) no-zero weight symmetric resampling frequencies.

The GSCT of all the most parsimonious trees (MPTs; figure 7*a*, table 1) found using the different concavity constant values (*k* = 5−8) in the modified Ezcurra *et al*. [40] matrix is very well-resolved and congruent with the consensus trees recovered in recent versions of this dataset (e.g. [7,40]). This consensus includes a taxonomically broad Herrerasauria composed of species from the middle Norian–Rhaetian of North America (*Tawa hallae*, *Chindesaurus bryansmalli*, TTU-P10082 and *Daemonosaurus chauliodus*) and the South American Carnian herrerasaurids. The new species *Maleriraptor kuttyi* is found within Herrerasauria because of the presence of an ilium with the distal extent of the supraacetabular crest ending well proximal to the pubic facet (character 190: 0→1, reversed in herrerasaurids), and more closely related to the South American herrerasaurids than to the *Tawa* group (i.e. *Tawa hallae*, *Chindesaurus bryansmalli* and *Daemonosaurus chauliodus*) because of the presence of a pubis ventrally or slightly posteroventrally oriented (character 204: 0→1). In particular, the North American specimen TTU-P10082 is found as the sister taxon of the South American herrerasaurids, and *Maleriraptor kuttyi* is excluded from this clade because it lacks a pubis with the lateral portion of the distal apron flipped posteriorly (character 391: 0→1). TTU-P10082 is excluded from Herrerasauridae because of the absence of an ilium with the distal extent of the supraacetabular crest extending up to the pubic facet (character 190: 1→0).

**Table 1.** Number of most parsimonious trees (MPTs) found and homoplasy indices of the four analyses of the modified Ezcurra *et al*. [40] matrix under implied weighting with the different concavity constant values.

concavity constant value (*k*)	number of MPTs	consistency index	retention index	fit (adjusted homoplasy)
5	3	0.34523	0.67510	105.38122
6	405	0.34548	0.67546	93.88208
7	405	0.34548	0.67546	84.70972
8	405	0.34548	0.67546	77.21068

The resampling frequencies are generally high throughout the tree (i.e. greater than 80%), but they are less than or equal to 52% in the clade composed of the North American herrerasaurs, the clade formed by *Maleriraptor kuttyi* and more deeply nested herrerasaurs, and the clade formed by TTU-P10082 + Herrerasauridae. The resampling frequencies of Herrerasauria are very high in the analyses under all *k*-values, ranging from 90% (absolute) and 87% (GC) under *k* = 5 to 96% (absolute) and 95% (GC) under *k* = 8. The frequencies of Herrerasauridae and the *Gnathovorax cabreirai + Herrerasaurus ischigualastensis* clade are very high under all *k*-values (i.e. greater than 94%).

The GSCT of all the MPTs found using the different concavity constant values (*k* = 3−10; table 2) in the modified Garcia *et al*. [19] matrix is considerably less resolved than the strict consensus tree recovered by these authors under equal weights. The GSCT of our study has large polytomies at the base of Avemetatarsalia and around the base of Dinosauria, and massive polytomies at the base of the ‘silesaurids + core ornithischians clade’ and Saurischia (see electronic supplementary material). The polytomy among early avemetatarsalians is partially a result of the alternative positions that *Faxinalipterus minimus* adopts at the base of Lagerpetidae, Dinosauromorpha or Dinosauriformes in the MPTs under *k* = 3 and 4. It is interesting to note that in these MPTs, Lagerpetidae is recovered as the sister taxon to a clade composed of Aphanosauria and Dinosauriformes. Nevertheless, aphanosaurs adopt their more traditional position at the base of Avemetatarsalia in the MPTs under *k* = 5−10, and *Faxinalipterus minimus* is found as the sister taxon to Ornithodira in these trees. The polytomy around the base of Dinosauria is because *Soumyasaurus aenigmaticus* is alternatively found as a non-dinosaurian dinosauriform, an early ornithischian (including silesaurids), or at the base of Saurischia. The unresolved relationships among the silesaurids result from the alternative positions of *Technosaurus smalli* among the MPTs. The polytomy at the base of Saurischia is the consequence of the alternative positions that *Tawa hallae*, *Chindesaurus bryansmalli* and CAPPA/UFSM 0373 adopt as a clade sister to ‘coelophysoid-grade’ theropods (*k* = 3−5) or among the earliest diverging members of Herrerasauria (*k* = 6−10).

**Table 2.** Number of MPTs found and homoplasy indices of the eight analyses of the modified Garcia *et al*. [19] matrix under implied weighting with the different concavity constant values.

concavity constant value (*k*)	number of MPTs	consistency index	retention index	fit (adjusted homoplasy)
3	324	0.289451	0.681142	124.20007
4	>100 000	0.29046	0.68271	106.72643
5	>100 000	0.29097	0.68349	93.72549
6	>100 000	0.29433	0.68858	83.60763
7	>100 000	0.29433	0.68858	75.43934
8	>100 000	0.29433	0.68858	68.75643
9	>100 000	0.29433	0.68858	63.18124
10	>100 000	0.29459	0.68897	58.45304

A monophyletic Herrerasauria minimally composed of *Maleriraptor kuttyi*, TTU-P10082 and herrerasaurids is recovered in all the MPTs under all *k*-values (figure 7*b*). In those MPTs in which CAPPA/UFSM 0373 and the clade composed of *Tawa hallae + Chindesaurus bryansmalli + Daemonosaurus chauliodus* are found among herrerasaurs, *Maleriraptor kuttyi* is recovered as a member of Herrerasauria because of the presence of an ilium with a markedly concave ventral margin of the acetabular wall (character 175: 1→2), a strong pillar posterior to the preacetabular embayment (character 180: 0→1), a maximum length of the postacetabular ala shorter than or subequal to the space between the pre- and post-acetabular embayments (character 183: 1→0), pubis ventrally or slightly posteroventrally oriented (mesopubic) (character 189: 0→1), and a pubic peduncle significantly more ventrally extended than the ischiadic peduncle (character 283: 0→1). In particular, *Maleriraptor kuttyi* is closer to herrerasaurids and the ‘*Tawa* clade’ than to CAPPA/UFSM 0373 because of an ilium without a brevis fossa (character 174: 1→0; scored as an embankment on the lateral surface of the postacetabular process in CAPPA/UFSM 0373 by Garcia *et al*. [19]). This latter character state is optimized as an additional synapomorphy of Herrerasauria in those MPTs in which CAPPA/UFSM 0373, *Tawa hallae*, *Chindesaurus bryansmalli* and *Daemonosaurus chauliodus* are recovered outside of Herrerasauria, as non-neotheropod theropods.

*Maleriraptor kuttyi* is excluded from the clade composed of TTU-P10082 + Herrerasauridae because of the absence of a pubic distal end expanded at least twice the breadth of the pubic shaft (character 190: 1→2) and pubis with posteriorly flipped lateral portion of the distal apron (character 192: 0→1). Moreover, *Maleriraptor kuttyi* lacks the following herrerasaurid synapomorphies: ilium with thicker (lateromedially) portion of the supraacetabular crest closer to pubic peduncle (character 172: 0→1, unknown in TTU-P10082) and ilium with supraacetabular crest extending along the pubic peduncle length (character 173: 0→1).

The resampling frequencies are relatively low (less than or equal to 59%) throughout the taxonomically broader Herrerasauria in the analysis under *k* = 6, but all the frequencies increase gradually with higher *k*-values (e.g. Herrerasauria absolute = 67% and GC = 41%, Herrerasauridae absolute = 70% and GC = 55% in the analysis under *k* = 10). The resampling frequencies of the more taxonomically limited Herrerasauria are relatively high under *k* = 3 (absolute = 75% and GC = 65%) but decrease gradually under *k* = 4 and 5 (absolute = 56% and GC = 33%). All the other frequencies within Herrerasauria are less than or equal to 65% under *k* = 3−5.

## 5. Discussion

### 5.1. The taxonomy of ISIR 282

The preserved sacral, caudal and pelvic girdle bones of the holotype of *Maleriraptor kuttyi* (ISIR 282) possess a unique combination of character states that distinguish this species from all other known early dinosauromorphs. The phylogenetic analyses show that *Maleriraptor kuttyi* can be included within Herrerasauria based on character states such as a short iliac postacetabular process, ilium without a brevis fossa, and an iliac pubic peduncle significantly more ventrally extended than the ischiadic peduncle. The very poorly anteroposteriorly expanded distal end of the pubis is an unexpected feature among herrerasaurs because all the other species have a well-developed distal pubic boot (e.g. [1,3,6,7]). The presence of a vertically oriented pubic shaft is shared between *Maleriraptor kuttyi* and herrerasaurids, but the Indian species lacks other features typical of the latter group, such as an iliac supraacetabular crest that reaches the distal end of the pubic peduncle [1,6]. Thus, the holotype of *Maleriraptor kuttyi* (ISIR 282) is clearly diagnostic at a species level.

Novas *et al*. [35], when first describing ISIR 282, recognized that this specimen was diagnostic at a species level based on a unique combination of character states. However, they refrained from erecting a new species because ISIR 282 lacked overlapping elements with the holotype and only known specimen of *Alwalkeria maleriensis* from the Lower Maleri Formation. Unfortunately, it is still impossible to compare the anatomy of *Alwalkeria maleriensis* and ISIR 282 directly. The absence of diagnostic features of lesser inclusive saurischian clades in the holotype of *Alwalkeria maleriensis*—a partial femur and an astragalus (the partial skull has been reinterpreted as belonging to an early crocodylomorph; Lecuona *et al*. [69])—led to the lack of consensus regarding its phylogenetic relationships, being alternatively classified as an early theropod (e.g. [70,71]) or an indeterminate saurischian (e.g. [5,35,54,72]). Thus, herrerasaurian affinities cannot be completely ruled out for *Alwalkeria maleriensis* and phylogeny does not inform if it is a different species to that of ISIR 282.

*Alwalkeria maleriensis* comes from the Lower Maleri Formation, which is the stratigraphic unit that underlies the Upper Maleri Formation that yielded the remains of ISIR 282. More importantly, the Lower Maleri Formation preserves a tetrapod assemblage numerically dominated by the hyperodapedontine rhynchosaur *Hyperodapedon huxleyi* [73]. This allows correlating biostratigraphically the Lower Maleri Formation with the *Hyperodapedon* Assemblage Zones/Biozones of other regions of Pangaea, such as the lower third of the Ischigualasto Formation of northwestern Argentina, the lower portion of the Candelária Sequence of the Santa Maria Supersequence of southern Brazil, the Pebbly Arkose of Zimbabwe and the Lossimouth Sandstone of Scotland [74]. In particular, dinosaur assemblages known from both *Hyperodapedon*-dominated and overlying rhynchosaur-free levels are known in the Santa Maria Supersequence of Brazil [8,75]—other stratigraphic sequences worldwide lack this faunistic transition or both assemblages are temporally more distant (e.g. the Ischigualasto Formation and the upper levels of the Los Colorados Formation). The *Hyperodapedon*-dominated assemblage of southern Brazil has a drastically different species-level composition to that of the younger levels [7,8]. Moreover, immediately overlying levels with the presence of the hyperodapedontine *Teyumbaita* have a tetrapod assemblage similar to that of the *Hyperodapedon* Assemblage Zone in both Brazil and Argentina [76,77]. Thus, it could be expected that the dinosaur assemblages between the *Hyperodapedon*-dominated Lower Maleri Formation and the rhynchosaur-free Upper Maleri Formation are also very different.

In conclusion, following the morphological uniqueness of ISIR 282 and the strong faunistic differences expected between the species-level composition of the Lower and Upper Maleri formations, here we erect the new species *Maleriraptor kuttyi*. Future discoveries of additional, more complete specimens of *Maleriraptor kuttyi* and/or *Alwalkeria maleriensis* would allow the first direct comparisons between the anatomy of these species and test the hypothesis proposed here that they belong to different taxa.

### 5.2. The taxonomic content and phylogenetic relationships of Herrerasauria

The phylogenetic data matrices used here to test the affinities of *Maleriraptor kuttyi* are modifications of those that have recently recovered a taxonomically broader Herrerasauria composed of the South American herrerasaurids and three younger species from North America (i.e. *Tawa hallae*, *Chindesaurus bryansmalli* and *Daemonosaurus chauliodus*) [7,19]. Here, we also recovered this topological arrangement in most of the analyses, but to the exclusion of the modified data matrix of Garcia *et al*. [19] analysed under *k*-values of 3−5. These three analyses strongly penalize the homoplasy [78] and decrease the weights of the character states that *Tawa hallae + Chindesaurus bryansmalli* share with herrerasaurids, favouring their position as the sister taxa to ‘coelophysoid-grade’ theropods instead. This latter position is that more traditionally recovered for *Tawa hallae* (e.g. [14,16,20,28,79]).

The branch supports of the taxonomically broad Herrerasauria increase with higher *k*-values in both data matrices, indicating that this clade is supported by characters with some degree of homoplasy. Nevertheless, the taxonomically broad Herrerasauria is more stable against homoplasy downweighting in the data matrix modified from Ezcurra *et al*. [40] because this clade persists even when the dataset is analysed under *k* = 3 and 4. *k*-values of 3 and 4 are probably penalizing too strongly the homoplasy in the matrix modified from Garcia *et al*. [19] because Ezcurra [41] found that these *k*-values clearly underperform higher *k*-values and even equal weights in the genealogy of the *‘Tawa* matrix’. This latter genealogy shares several taxa and characters with the Garcia *et al*. [19] matrix, and hence, it is likely that they behave similarly under homoplasy downweighting. Thus, it is very likely that *k*-values of 3 and 4 are penalizing homoplasy too strongly in the Garcia *et al*. [19] matrix. Moreover, the Garcia *et al*. [19] matrix has more terminals than that of Ezcurra *et al*. [40] and the range of *k*-values that could produce more stable results through the genealogy is probably displaced towards higher values than in the latter [41]. Thus, we consider that the hypothesis of a taxonomically broader Herrerasauria (i.e. including *Tawa hallae* and *Chindesaurus bryansmalli*) is the most reliable based on the two datasets analysed here. Another fact in favour of the taxonomically broader Herrerasauria hypothesis is that in the first versions of the Complete Archosauromorph Tree Project (CoArTreeP; see [41,80]) that included *Tawa hallae*, this species was recovered as the sister taxon to Neotheropoda [79,81]. However, *Tawa hallae* is recovered as the sister taxon to the herrerasaurids in the more recent iterations of this matrix, which have a larger taxon and character sampling [82,83]. Nevertheless, the breakage of the monophyly of the taxonomically broad Herrerasauria under strong homoplasy penalization in the modified Garcia *et al*. [19] matrix is a warning flag that this hypothesis is not very robust in that dataset, and more work should be conducted on this topic.

The North American specimen TTU-P10082 was originally interpreted as a saurischian similar to the herrerasaurid *Staurikosaurus* [25] and more recently as a herrerasaurid [26]. Here, the affinities of this specimen were tested for the first time in a quantitative phylogeny, and it was recovered in both analyses as a non-herrerasaurid herrerasaurian. Nesbitt & Chatterjee [25] considered that TTU-P10082 was diagnostic at a species level but refrained from naming it because it could belong to another, already-named taxon without or with very limited overlapping bones, such as *Chindesaurus bryansmalli*. The results of our phylogenetic analyses showed that, although both are non-herrerasaurid herrerasaurs, *Chindesaurus bryansmalli* and TTU-P10082 are not sister taxa to each other. Indeed, either TTU-P10082 or the *Chindesaurus + Tawa* clade are recovered alternatively as more closely related to herrerasaurids in the different analyses. These results favour the hypothesis that TTU-P10082 could belong to a yet unnamed North American herrerasaur species, different at least from those nominal species recorded in the Chinle Formation. Nevertheless, we also refrain from erecting a new taxon for TTU-P10082 because we cannot confidently distinguish it from the holotype of *Caseosaurus crosbyensis*, also from the Dockum Group of Texas, which is represented by a fairly complete, isolated right ilium [12,18,84]. The herrerasaurian affinities of *Caseosaurus crosbyensis*, and thus at least a close relationship to TTU-P10082, are supported by a short postacetabular process without a brevis fossa, a pubic peduncle considerably more ventrally extended than the ischiadic one, and a rib for the first primordial sacral rib reaching ventrally the base of the pubic peduncle, indicating a dorsoventrally very tall contact with the ilium [1,19,55].

The higher level phylogenetic relationships of Herrerasauria have been long debated, being alternatively interpreted as non-dinosaurian dinosauriforms [1,18], non-eusaurischian saurischians (e.g. [3,6,7,10,19,24,54,55,61,82,85,86]), non-neotheropod theropods (e.g. [14,23,79,87,88]) or even as the earliest diverging sauropodomorphs [17]. The analyses of the two phylogenetic datasets used here agree with the position of the herrerasaurs as non-eusaurischian saurischians. The resampling frequencies of Saurischia are very high, with mean values (average of all *k*-values) of 100% (absolute and GC) in the modified Ezcurra *et al*. [40] matrix and 91% (absolute) and 89% (GC) in the modified Garcia *et al*. [19] matrix. Hence, the position of Herrerasauria as saurischians is very robust in these datasets. By contrast, the frequencies of Eusaurischia are lower than 50% in the modified Garcia *et al*. [19] matrix, even in those analyses in which *Tawa hallae* and *Chindesaurus bryansmalli* are recovered as theropods, and lower than 69% in the modified Ezcurra *et al*. [40] matrix in all cases. Thus, although the non-eusaurischian saurischian position of herrerasaurs seems to be gaining consensus among recent independent studies (see also Müller *et al*. [82] for the CoArTreeP matrix), these low branch supports also indicate that more work is needed in this part of the early dinosaur tree.

### 5.3. The evolution of the pubic boot in Herrerasauria

One of the most striking features of *Maleriraptor kuttyi* is the absence of the very well-developed pubic boot that characterizes herrerasaurs (e.g. [1,3,6,7]). If it is considered the phylogenetic hypothesis in which *Maleriraptor kuttyi* is positioned at the base of Herrerasauria (Garcia *et al*. [19] modified matrix under *k* = 3−5) or it is the earliest diverging herrerasaur to the exclusion of CAPPA/UFSM 0373 (Garcia *et al*. [19] modified matrix under *k* = 6−10), the poorly expanded distal end of the pubis would represent the ancestral condition of Herrerasauria. However, *Maleriraptor kuttyi* is more deeply nested within Herrerasauria in the analyses of the matrix modified from Ezcurra *et al*. [40], and it is bracketed by species with well-developed pubic boots (i.e. *Tawa hallae* and TTU-P10082 + herrerasaurids, respectively). Thus, the discovery of *Maleriraptor kuttyi* complicates the interpretation of the evolution of the herrerasaurian pubic boot because there are two equally parsimonious optimizations of this character in the latter analyses. One possibility is that the pubic boot was independently acquired in *Tawa hallae* and the TTU-P10082 + herrerasaurids clade, and the other is that its absence is an apomorphy of *Maleriraptor kuttyi*. A well-developed pubic boot has been acquired independently at least three times among Triassic–Early Jurassic archosaurs: paracrocodylomorph pseudosuchians, herrerasaurs and averostran theropods [57], whereas the condition has been lost subsequently in crocodylomorphs within Paracrocodylomorpha [57] and parvicursorine alvarezsaurids and ornithurine birds within Theropoda [89–91]. Despite the function of the pubic boot being far from being well understood, its occurrence has been correlated with an increased surface for the attachment of the abdominal muscles (i.e. *M. rectus abdominus, M. obliquus abdominus* and *M. ischiocaudalis*), the site of origin for suprapubic musculature [92,93], or as a guide for the ischiotruncus muscle [94], as well as the anchoring of the pelvic medial membrane [92]. The pubic boot becomes reduced in the line to birds, correlated with pubic retrovertion and concomitant modifications of the abdominal musculature and loss of cuirassal breathing [92,94]. Nevertheless, the acquisition and loss of a well-developed pubic boot seems to have some degree of evolutionary plasticity among archosaurs, and it could have also been the case within Herrerasauria. Future efforts should focus on a more detailed comparison to evaluate the primary homology between the pubic boot of the earliest diverging herrerasaurs and herrerasaurids to determine if they are independent acquisitions.

### 5.4. The implications of *Maleriraptor kuttyi* for the spatio-temporal distribution of Herrerasauria

**Figure 8.**
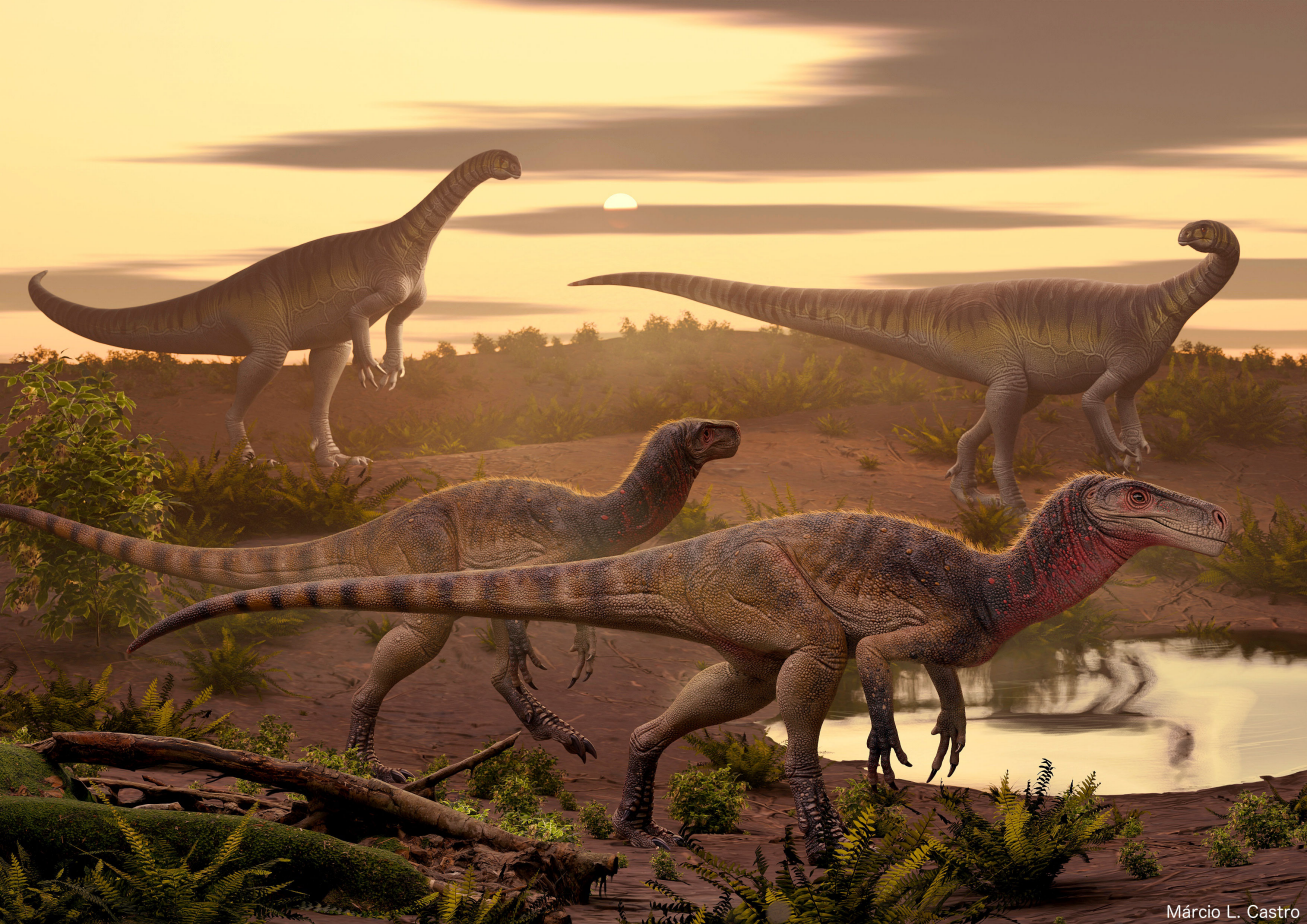
Life reconstruction of *Maleriraptor kuttyi* with the unaysaurid sauropodomorph *Jaklapallisaurus asymmetricus*, both from the lower Norian Upper Maleri Formation of south-central India. Artwork by Márcio L. Castro.

South American herrerasaurs are restricted to *Hyperodapedon*-dominated beds of the Ischigualasto Formation and the Santa Maria Supersequence [6,8,11,19] dated as middle Carnian–lowermost Norian (*ca* 233−229 Ma; [8,9]). More recently, an indeterminate herrerasaur from the Pebbly Arkose Formation of Zimbabwe, which is considered approximately coeval to the above-mentioned South American units because of the abundance of hyperodapedontine rhynchosaurs in its assemblage [74], expanded the record of the clade into the south of the African continent [30]. By contrast, the North American and European herrerasaur records are middle Norian to Rhaetian in age [7,12,18,19,26,29], in which the Chinle Formation specimens are younger than *ca* 213 Ma (Petrified Forest Member–Sonsela Member contact [95]; and the Dockum Group specimens are probably younger than 220 Ma (Post Quarry age; [96]). Thus, there was a temporal gap between the records of the Southern and Northern hemispheres of approximately 9 Myr and it seemed that the southern herrerasaurs were one of the victims of the earliest Norian terrestrial faunistic turnover that included the worldwide extinction of the rhynchosaurs. The discovery of *Maleriraptor kuttyi* shows that herrerasaurs survived in Gondwana at least during the early Norian after the event that vanished the rhynchosaurs. The presence of herrerasaurs in the early Norian of India and not in South America could be climatically related because global palaeoclimatic reconstructions indicate that India had mean annual temperatures and precipitations more similar to those of southern North America in the Norian [97]. Thus, the more similar palaeoclimate between India and southern North America can explain the presence of common faunistic components that are absent in south-central South America (or are extremely rare), such as phytosaurs, herrerasaurs, protopyknosians and malerisaurine allokotosaurs [98,99]. The deposition of the Upper Maleri Formation probably occurred shortly after the extinction of rhynchosaurs, which are abundantly recorded in the Lower Maleri Formation. Faunistic resemblances between the Upper Maleri Formation and the upper section of the Santa Maria Supersequence of Brazil, such as the presence of unaysaurids ([37]; figure 8), suggest a similar age that it is dated in *ca* 225 Ma in the Brazilian unit [8]. Thus, *Maleriraptor kuttyi* partially fills the early Norian gap in the herrerasaur record.

## Data Availability

Both phylogenetic data matrices in TNT and Nexus formats, their character lists, a TNT script to conduct the phylogenetic analyses, and their results have been uploaded in a single compressed electronic supplementary material file accompanying this article [100].

## References

[1] Novas FE. 1992 Phylogenetic relationships of the basal dinosaurs, the Herrerasauridae. Palaeontology **35**, 51–62.

[2] Novas F. 1997 Herrerasauridae. In Encyclopedia of dinosaurs (eds P Currie, K Padian), pp. 303–311. San Diego, CA: Academic Press.

[3] Martínez RN, Alcober OA. 2010 A new herrerasaurid (Dinosauria, Saurischia) from the Upper Triassic Ischigualasto Formation of northwestern Argentina. Zookeys **63**, 55–81. (10.3897/zookeys.63.550) PMC3088398 21594020

[4] Brusatte SL, Nesbitt SJ, Irmis RB, Butler RJ, Benton MJ, Norell MA. 2010 The origin and early radiation of dinosaurs. Earth Sci. Rev. **101**, 68–100. (10.1016/j.earscirev.2010.04.001)

[5] Langer MC, Ezcurra MD, Bittencourt JS, Novas FE. 2010 The origin and early evolution of dinosaurs. Biol. Rev. **85**, 55–110. (10.1111/j.1469-185x.2009.00094.x) 19895605

[6] Pacheco C, Müller RT, Langer M, Pretto FA, Kerber L, Dias da Silva S. 2019 Gnathovorax cabreirai: a new early dinosaur and the origin and initial radiation of predatory dinosaurs. PeerJ **7**, e7963. (10.7717/peerj.7963) 31720108 PMC6844243

[7] Novas FE, Agnolín FL, Ezcurra MD, Temp Müller R, Martinelli AG, Langer MC. 2021 Review of the fossil record of early dinosaurs from South America, and its phylogenetic implications. J. South Am. Earth Sci. **110**, 103341. (10.1016/j.jsames.2021.103341)

[8] Langer MC, Ramezani J, Da Rosa ÁAS. 2018 U-Pb age constraints on dinosaur rise from south Brazil. Gondwana Res. **57**, 133–140. (10.1016/j.gr.2018.01.005)

[9] Colombi C, Martínez RN, Césari SN, Alcober O, Limarino CO, Montañez I. 2021 A high-precision U–Pb zircon age constraints the timing of the faunistic and palynofloristic events of the Carnian Ischigualasto Formation, San Juan, Argentina. J. South Am. Earth Sci. **111**, 103433. (10.1016/j.jsames.2021.103433)

[10] Garcia MS, Müller RT, Pretto FA, Da-Rosa ÁAS, Dias-Da-Silva S. 2021 Taxonomic and phylogenetic reassessment of a large-bodied dinosaur from the earliest dinosaur-bearing beds (Carnian, Upper Triassic) from southern Brazil. J. Syst. Palaeontol. **19**, 1–37. (10.1080/14772019.2021.1873433)

[11] Martínez RN, Apaldetti C, Alcober OA, Colombi CE, Sereno PC, Fernandez E, Malnis PS, Correa GA, Abelin D. 2012 Vertebrate succession in the Ischigualasto Formation. J. Vertebr. Paleontol. **32**, 10–30. (10.1080/02724634.2013.818546)

[12] Long R, Murry P. 1995 Late Triassic (Carnian and Norian) tetrapods from the southwestern United States. N. M. Mus. Nat. Hist. Sci. Bull. **4**, 1–254.

[13] Irmis RB, Nesbitt SJ, Padian K, Smith ND, Turner AH, Woody D, Downs A. 2007 A Late Triassic dinosauromorph assemblage from New Mexico and the rise of dinosaurs. Science **317**, 358–361. (10.1126/science.1143325) 17641198

[14] Nesbitt SJ, Smith ND, Irmis RB, Turner AH, Downs A, Norell MA. 2009 A complete skeleton of a Late Triassic saurischian and the early evolution of dinosaurs. Science **326**, 1530–1533. (10.1126/science.1180350) 20007898

[15] Ezcurra MD, Brusatte SL. 2011 Taxonomic and phylogenetic reassessment of the early neotheropod dinosaur Camposaurus arizonensis from the Late Triassic of North America. Palaeontology **54**, 763–772. (10.1111/j.1475-4983.2011.01069.x)

[16] Sues HD, Nesbitt SJ, Berman DS, Henrici AC. 2011 A late-surviving basal theropod dinosaur from the latest Triassic of North America. Proc. R. Soc. B **278**, 3459–3464. (10.1098/rspb.2011.0410) PMC3177637 21490016

[17] Baron MG, Norman DB, Barrett PM. 2017 A new hypothesis of dinosaur relationships and early dinosaur evolution. Nature **543**, 501–506. (10.1038/nature21700) 28332513

[18] Baron MG, Williams ME. 2018 A re-evaluation of the enigmatic dinosauriform Caseosaurus crosbyensis from the Late Triassic of Texas, USA and its implications for early dinosaur evolution. Acta Palaeontol. Pol. **63**, 129–145. (10.4202/app.00372.2017)

[19] Garcia MS, Cabreira SF, da Silva LR, Pretto FA, Müller RT. 2024 A saurischian (Archosauria, Dinosauria) ilium from the Upper Triassic of southern Brazil and the rise of Herrerasauria. Anat. Rec. **307**, 1011–1024. (10.1002/ar.25342) 37971103

[20] Nesbitt SJ, Ezcurra MD. 2015 The early fossil record of dinosaurs in North America: a new neotheropod from the base of the Upper Triassic Dockum Group of Texas. Acta Palaeontol. Pol. **60**, 513–526. (10.4202/app.00143.2014)

[21] Langer MC, Ezcurra MD, Rauhut OWM, Benton MJ, Knoll F, McPhee BW, Novas FE, Pol D, Brusatte SL. 2017 Untangling the dinosaur family tree. Nature **551**, E1–E3. (10.1038/nature24011) 29094688

[22] Baron MG, Norman DB, Barrett PM. 2017 Baron et al. reply. Nature **551**, E4–E5. (10.1038/nature24012) 29094705

[23] Marsh AD, Parker WG, Langer MC, Nesbitt SJ. 2019 Redescription of the holotype specimen of Chindesaurus bryansmalli Long and Murry, 1995 (Dinosauria, Theropoda), from Petrified Forest National Park, Arizona. J. Vertebr. Paleontol. **39**, e1645682. (10.1080/02724634.2019.1645682)

[24] Norman DB FLS, Baron MG, Garcia MS, Müller RT. 2022 Taxonomic, palaeobiological and evolutionary implications of a phylogenetic hypothesis for Ornithischia (Archosauria: Dinosauria). Zool. J. Linn. Soc. **196**, 1273–1309. (10.1093/zoolinnean/zlac062)

[25] Nesbitt SJ, Chatterjee S. 2008 Late Triassic dinosauriforms from the Post Quarry and surrounding areas, west Texas, USA. Neues Jahrb. Für Geol. Und Paläontol. Abh. **252**, 143–156. (10.1127/0077-7749/2008/0249-0143)

[26] Sarıgül V. 2017 New theropod fossils from the Upper Triassic Dockum Group of Texas, USA, and a brief overview of the Dockum theropod diversity. PaleoBios **34**, 1–18. (10.5070/P9341033817)

[27] Nesbitt SJ, Langer MC, Ezcurra MD. 2019 The anatomy of Asilisaurus kongwe, a dinosauriform from the Lifua Member of the Manda Beds (~Middle Triassic) of Africa. Anat. Rec. **303**, 813–873. (10.1002/ar.24287) 31797580

[28] Nesbitt SJ, Sues HD. 2021 The osteology of the early-diverging dinosaur Daemonosaurus chauliodus (Archosauria: Dinosauria) from the Coelophysis Quarry (Triassic: Rhaetian) of New Mexico and its relationships to other early dinosaurs. Zool. J. Linn. Soc. **191**, 150–179. (10.1093/zoolinnean/zlaa080)

[29] Niedźwiedzki G, Brusatte S, Sulej T, Butler R. 2014 Basal dinosauriform and theropod dinosaurs from the Mid–Late Norian (Late Triassic) of Poland: implications for Triassic dinosaur evolution and distribution. Palaeontology **57**, 1121–1142.

[30] Griffin CT, Wynd BM, Munyikwa D, Broderick TJ, Zondo M, Tolan S, Langer MC, Nesbitt SJ, Taruvinga HR. 2022 Africa’s oldest dinosaurs reveal early suppression of dinosaur distribution. Nature **609**, 313–319. (10.1038/s41586-022-05133-x) 36045297

[31] Novas F, Chatterjee S, Ezcurra M, Kutty T. 2009 New dinosaur remains from the Late Triassic of Central India. J. Vertebr. Paleontol. **29**, 156A.

[32] Ray S, Bhat MohdS, Mukherjee D, Datta PM. 2016 Vertebrate fauna from the Late Triassic Tiki Formation of India: new finds and their biostratigraphic implications. J. Palaeosciences **65**, 47–59. (10.54991/jop.2016.298)

[33] Sengupta S, Ezcurra MD, Bandyopadhyay S. 2017 A new horned and long-necked herbivorous stem-archosaur from the Middle Triassic of India. Sci. Rep. **7**, 1–9. (10.1038/s41598-017-08658-8) 28827583 PMC5567049

[34] Kutty T, Sengupta D. 1989 The Late Triassic formations of the Pranhita-Godavari Valley and their vertebrate faunal succession—a reappraisal. Indian J. Earth Sci. **16**, 189–206.

[35] Novas FE, Ezcurra MD, Chatterjee S, Kutty TS. 2010 New dinosaur species from the Upper Triassic Upper Maleri and Lower Dharmaram formations of central India. Earth Environ. Sci. Trans. R. Soc. Edinb. **101**, 333–349. (10.1017/S1755691011020093)

[36] Sarma B, Krishna Rao M. 2005 Basement structure of Godavari basin, India–geophysical modelling. Curr. Sci. **88**, 1172–1175.

[37] Ezcurra MD, Müller RT, Novas FE, Chatterjee S. 2024 Osteology of the sauropodomorph dinosaur Jaklapallisaurus asymmetricus from the Late Triassic of central India. Anat. Rec. **307**, 1093–1112. (10.1002/ar.25359) 38088472

[38] Kutty T, Jain S, Chowdhury TR. 1987 Gondwana sequence of the northern Pranhita–Godavari Valley: its stratigraphy and vertebrate faunas. Palaeobot **36**, 214–219. (10.54991/jop.1987.1582)

[39] Kutty TS, Chatterjee S, Galton PM, Upchurch P. 2007 Basal sauropodomorphs (Dinosauria: Saurischia) from the Lower Jurassic of India: their anatomy and relationships. J. Paleontol. **81**, 1218–1240. (10.1666/04-074.1)

[40] Ezcurra MD, Marke D, Walsh SA, Brusatte SL. 2023 A revision of the ‘coelophysoid-grade’ theropod specimen from the Lower Jurassic of the Isle of Skye (Scotland). Scott. J. Geol. **59** 1–10. (10.1144/sjg2023-012)

[41] Ezcurra MD. 2024 Exploring the effects of weighting against homoplasy in genealogies of palaeontological phylogenetic matrices. Cladistics **40**, 242–281. (10.1111/cla.12581) 38728134

[42] Goloboff PA, Morales ME. 2023 TNT version 1.6, with a graphical interface for MacOS and Linux, including new routines in parallel. Cladistics **39**, 144–153. (10.1111/cla.12524) 36682054

[43] Goloboff PA, Torres A, Arias JS. 2018 Weighted parsimony outperforms other methods of phylogenetic inference under models appropriate for morphology. Cladistics **34**, 407–437. (10.1111/cla.12205) 34649370

[44] Swofford D, Begle D. 1993 PAUP: phylogenetic analysis using parsimony. User’s manual. Champaign, IL: Illinois Natural History Survey.

[45] Coddington JA, Scharff N. 1994 Problems with zero‐length branches. Cladistics **10**, 415–423. (10.1111/j.1096-0031.1994.tb00187.x)

[46] Spiekman SNF, Ezcurra MD, Butler RJ, Fraser NC, Maidment SCR. 2021 Pendraig milnerae, a new small-sized coelophysoid theropod from the Late Triassic of Wales. R. Soc. Open Sci. **8**, 210915. (10.1098/rsos.210915) 34754500 PMC8493203

[47] Bona P, Barrios F, Ezcurra MD, Fernandez Blanco MV, Cidade GM. 2024 New taxa of giant caimans from the southernmost hyperdiverse wetlands of the South American late Miocene. J. Syst. Palaeontol. **22**, 2375027. (10.1080/14772019.2024.2375027)

[48] Ezcurra MD, Fernandes AE, Roig M, Von Baczko MB. 2025 A revision of the pterodactyloid pterosaur Herbstosaurus pigmaeus Casamiquela, 1975 from the Late Jurassic of Argentina. An. Acad. Bras. Ciênc. **97**, e20241130. (10.1590/0001-3765202520241130) 40008776

[49] Owen R. 1842 Report on British fossil reptiles, Part II. Rep. Br. Assoc. Adv. Sci. **11**, 60–204.

[50] Langer M, Bittencourt J, Novas F, Ezcurra M. 2020 Dinosauria. In Phylonyms: a companion to the phylocode (eds K de Queiroz, P Cantino, J Gauthier), pp. 1209–1217. Boca Raton, FL: CRC Press.

[51] Seeley H. 1888 On the classification of the fossil animals commonly named Dinosauria. Proc. R. Soc. Lond. **43**, 165–171. (10.1098/rspl.1887.0117)

[52] Gauthier J, Langer M, Bittencourt J, Novas F, Ezcurra M. 2020 Saurischia. In Phylonyms: a companion to the phylocode (eds K de Queiroz, P Cantino, J Gauthier), pp. 1219–1224. Boca Raton, FL: CRC Press.

[53] Galton PM. 1985 Notes on the Melanorosauridae, a family of large prosauropod dinosaurs (Saurischia: Sauropodomorpha). Geobios **18**, 671–676. (10.1016/s0016-6995(85)80065-6)

[54] Langer MC. 2004 Basal Saurischia. In The Dinosauria (eds DB Weishampel, P Dodson, H Osmólska), pp. 25–46, 2nd edn. Berkeley, CA: University of California Press. (10.1525/california/9780520242098.003.0004)

[55] Langer MC, Benton MJ. 2006 Early dinosaurs: a phylogenetic study. J. Syst. Palaeontol. **4**, 309–358. (10.1017/S1477201906001970)

[56] Novas F. 1993 New information on the systematics and postcranial skeleton of Herrerasaurus ischigualastensis (Theropoda: Herrerasauridae) from the Ischigualasto Formation (Upper Triassic) of Argentina. J. Vertebr. Paleontol. **13**, 400–423.

[57] Nesbitt SJ. 2011 The early evolution of archosaurs: relationships and the origin of major clades. Bull. Am. Mus. Nat. Hist. **352**, 1–292. (10.1206/352.1)

[58] De Souza Bittencourt J, Kellner AWA. 2009 The anatomy and phylogenetic position of the Triassic dinosaur Staurikosaurus pricei Colbert, 1970. Zootaxa **2079**, 1–56. (10.11646/zootaxa.2079.1.1)

[59] Dzik J. 2003 A beaked herbivorous archosaur with dinosaur affinities from the early Late Triassic of Poland. J. Vertebr. Paleontol. **23**, 556–574. (10.1671/a1097)

[60] Langer M. 2003 The pelvic and hind limb anatomy of the stem-sauropodomorph Saturnalia tupiniquim (Late Triassic, Brazil). PaleoBios **23**, 1–40.

[61] Ezcurra MD. 2010 A new early dinosaur (Saurischia: Sauropodomorpha) from the Late Triassic of Argentina: a reassessment of dinosaur origin and phylogeny. J. Syst. Palaeontol. **8**, 371–425. (10.1080/14772019.2010.484650)

[62] Sereno PC, Arcucci AB. 1993 Dinosaurian precursors from the Middle Triassic of Argentina: Lagerpeton chanarensis. J. Vertebr. Paleontol. **13**, 385–399. (10.1080/02724634.1994.10011522)

[63] Sereno PC, Arcucci AB. 1994 Dinosaurian precursors from the Middle Triassic of Argentina: Marasuchus lilloensis, gen. nov. J. Vertebr. Paleontol. **14**, 53–73. (10.1080/02724634.1994.10011538)

[64] Colbert E. 1970 A saurischian dinosaur from the Triassic of Brazil. Am. Mus. Novit. **2405**, 1–39.

[65] Bonaparte J, Ferigolo J, Ribeiro A. 1999 A new Late Triassic saurischian dinosaur from Rio Grande do Sul State, Brazil (eds Y Tomida, T Rich, P Vickers-Rich). In Proc. of the 2nd Gondwana Dinosaur Symp., vol. 15, pp. 89–109, Tokyo: National Science Museum.

[66] Bonaparte JF, Brea G, Schultz CL, Martinelli AG. 2007 A new specimen of Guaibasaurus candelariensis (basal Saurischia) from the Late Triassic Caturrita Formation of southern Brazil. Hist. Biol. **19**, 73–82. (10.1080/08912960600866862)

[67] Sullivan R, Lucas S. 1999 Eucoelophysis baldwini a new theropod dinosaur from the Upper Triassic of New Mexico, and the status of the original types of Coelophysis. J. Vertebr. Paleontol. **19**, 81–90.

[68] Reig O. 1963 La presencia de dinosaurios saurisquios en los ‘estratos de Ischigualasto’ (Mesotriasico Superior) de las provincias de San Juan y La Rioja (Argentina). Ameghiniana **3**, 3–20.

[69] Lecuona A, Ezcurra MD, Irmis RB. 2016 Revision of the early crocodylomorph Trialestes romeri (Archosauria, Suchia) from the lower Upper Triassic Ischigualasto Formation of Argentina: one of the oldest‐known crocodylomorphs. Pap. Palaeontol. **2**, 585–622. (10.1002/spp2.1056)

[70] Chatterjee S. 1987 A new theropod dinosaur from India with remarks on the Gondwana–Laurasia connection in the Late Triassic. In Gondwana 6: stratigraphy, sedimentology and paleontology, geophysical monograph (ed. G McKenzie), pp. 183–189, vol. 41. Washington, DC: American Geophysical Union.

[71] Khosla A, Lucas SG. 2024 Triassic-Jurassic dinosaurs from India, their ages and palaeobiogeographic significance. Hist. Biol. 1–26. (10.1080/08912963.2024.2336992)

[72] Remes K, Rauhut OWM. 2005 The oldest Indian dinosaur *Alwalkeria maleriensis* Chatterjee revised: a chimera including remains of a basal saurischian. In Boletim de resumos do ii congresso latino-americano de paleontologia de vertebrados (eds A Kellner, DDR Henriques, T Rodrigues), p. 218. Rio de Janeiro, Brazil: Museu Nacional / UFRJ.

[73] Chatterjee S. 1974 A rhynchosaur from the Upper Triassic Maleri Formation of India. Phil. Trans. R. Soc. Lond. B **267**, 209–261. (10.1098/rstb.1974.0001) 4149134

[74] Langer MC. 2005 Studies on continental Late Triassic tetrapod biochronology. II. The Ischigualastian and a Carnian global correlation. J. South Am. Earth Sci. **19**, 219–239. (10.1016/j.jsames.2005.04.002)

[75] Müller RT, Langer MC, Dias-da-Silva S. 2018 An exceptionally preserved association of complete dinosaur skeletons reveals the oldest long-necked sauropodomorphs. Biol. Lett. **14**, 20180633. (10.1098/rsbl.2018.0633) 30463923 PMC6283919

[76] Doering M, Ezcurra MD, Schiefelbein JH, Garcia MS, Müller RT. 2024 New archosauromorph remains provide data on the age of a unique Late Triassic assemblage from southern Brazil. J. South Am. Earth Sci. **145**, 105046. (10.1016/j.jsames.2024.105046)

[77] Desojo JB *et al*. 2020 The Late Triassic Ischigualasto Formation at Cerro Las Lajas (La Rioja, Argentina): fossil tetrapods, high-resolution chronostratigraphy, and faunal correlations. Sci. Rep. **10**, 12782. (10.1038/s41598-020-67854-1) 32728077 PMC7391656

[78] Goloboff PA. 1993 Estimating character weights during tree search. Cladistics **9**, 83–91. (10.1006/clad.1993.1003) 34929936

[79] Ezcurra MD *et al*. 2020 Enigmatic dinosaur precursors bridge the gap to the origin of Pterosauria. Nature **588**, 445–449. (10.1038/s41586-020-3011-4) 33299179

[80] Ezcurra MD. 2016 The phylogenetic relationships of basal archosauromorphs, with an emphasis on the systematics of proterosuchian archosauriforms. PeerJ **4**, e1778. (10.7717/peerj.1778) 27162705 PMC4860341

[81] Ezcurra MD, Bandyopadhyay S, Sengupta DP, Sen K, Sennikov AG, Sookias RB, Nesbitt SJ, Butler RJ. 2023 A new archosauriform species from the Panchet Formation of India and the diversification of Proterosuchidae after the end-Permian mass extinction. R. Soc. Open Sci. **10**, 230387. (10.1098/rsos.230387) 37885992 PMC10598453

[82] Müller RT, Ezcurra MD, Garcia MS, Agnolín FL, Stocker MR, Novas FE, Soares MB, Kellner AWA, Nesbitt SJ. 2023 New reptile shows dinosaurs and pterosaurs evolved among diverse precursors. Nature **620**, 589–594. (10.1038/s41586-023-06359-z) 37587301

[83] Sengupta S, Ezcurra MD, Bandyopadhyay S. 2024 The redescription of Malerisaurus robinsonae (Archosauromorpha: Allokotosauria) from the Upper Triassic Lower Maleri Formation, Pranhita‐Godavari Basin, India. Anat. Rec. **307**, 1315–1365. (10.1002/ar.25392) 38278769

[84] Hunt AP, Lucas SG, Heckert AB, Sullivan RM, Lockley MG. 1998 Late Triassic dinosaurs from the western United States. Geobios **31**, 511–531. (10.1016/s0016-6995(98)80123-x)

[85] Ezcurra M. 2006 A review of the systematic position of the dinosauriform archosaur Eucoelophysis baldwini Sullivan & Lucas, 1999 from the Upper Triassic of New Mexico, USA. Geodiversitas **28**, 649–684.

[86] Marsola JCA, Bittencourt JS, Butler RJ, Da Rosa ÁAS, Sayão JM, Langer MC. 2018 A new dinosaur with theropod affinities from the Late Triassic Santa Maria Formation, south Brazil. J. Vertebr. Paleontol. **38**, e1531878. (10.1080/02724634.2018.1531878)

[87] Novas F. 1996 Dinosaur monophyly. J. Vertebr. Paleontol. **16**, 723–741.

[88] Ezcurra MD. 2017 A new early coelophysoid neotheropod from the Late Triassic of northwestern Argentina. Ameghiniana **54**, 506–538. (10.5710/AMGH.04.08.2017.3100)

[89] Perle A, Chiappe L, Rinchen B, Clark J, Norell M, Akademi M. 1994 Skeletal morphology of Mononykus olecranus (Theropoda, Avialae) from the Late Cretaceous of Mongolia. Am. Mus. Novit. **3105**, 1–29.

[90] Novas F. 1996 Alvarezsauridae, Cretaceous basal birds from Patagonia and Mongolia. Memoirs Qld. Mus. **39**, 675–702.

[91] Hutchinson JR, Chiappe LM. 1998 The first known alvarezsaurid (Theropoda: Aves) from North America. J. Vertebr. Paleontol. **18**, 447–450. (10.1080/02724634.1998.10011073)

[92] Hutchinson JR. 2001 The evolution of pelvic osteology and soft tissues on the line to extant birds (Neornithes). Zool. J. Linn. Soc. **131**, 123–168. (10.1111/j.1096-3642.2001.tb01313.x)

[93] Ruben JA, Jones TD, Geist NR. 2003 Respiratory and reproductive paleophysiology of dinosaurs and early birds. Physiol. Biochem. Zool. **76**, 141–164. (10.1086/375425) 12794669

[94] Carrier DR, Farmer CG. 2000 The evolution of pelvic aspiration in archosaurs. Paleobiology **26**, 271–293. (10.1666/0094-8373(2000)026<0271:TEOPAI>2.0.CO;2)

[95] Ramezani J, Hoke GD, Fastovsky DE, Bowring SA, Therrien F, Dworkin SI, Atchley SC, Nordt LC. 2011 High-precision U-Pb zircon geochronology of the Late Triassic Chinle Formation, Petrified Forest National Park (Arizona, USA): temporal constraints on the early evolution of dinosaurs. Geol. Soc. Am. Bull. **123**, 2142–2159. (10.1130/B30433.1)

[96] Marsh AD, Parker WG. 2020 New dinosauromorph specimens from Petrified Forest National Park and a global biostratigraphic review of Triassic dinosauromorph body fossils. PaleoBios **37** 1–56. (10.5070/p9371050859)

[97] Dunne EM, Farnsworth A, Greene SE, Lunt DJ, Butler RJ. 2021 Climatic drivers of latitudinal variation in Late Triassic tetrapod diversity. Palaeontology **64**, 101–117. (10.1111/pala.12514)

[98] Nesbitt SJ, Stocker MR, Chatterjee S, Horner JR, Goodwin MB. 2021 A remarkable group of thick‐headed Triassic period archosauromorphs with a wide, possibly Pangean distribution. J. Anat. **239**, 184–206. (10.1111/joa.13414) 33660262 PMC8197959

[99] Nesbitt SJ *et al*. 2022 Widespread azendohsaurids (Archosauromorpha, Allokotosauria) from the Late Triassic of western USA and India. Pap. Palaeontol **8**, e1413. (10.1002/spp2.1413)

[100] Ezcurra MD, Garcia MS, Novas F, Müller RT, Agnolin FL, Chatterjee S. 2025 Supplementary material from: A new herrerasaurian dinosaur from the Upper Triassic Upper Maleri Formation of south-central India. Figshare. (10.6084/m9.figshare.c.7766565) PMC12077243 40370605

